# cAMP and EPAC Are Key Players in the Regulation of the Signal Transduction Pathway Involved in the α-Hemolysin Autophagic Response

**DOI:** 10.1371/journal.ppat.1002664

**Published:** 2012-05-24

**Authors:** María Belén Mestre, María Isabel Colombo

**Affiliations:** Laboratorio de Biología Celular y Molecular- Instituto de Histología y Embriología (IHEM), Facultad de Ciencias Médicas, Universidad Nacional de Cuyo-CONICET, Mendoza, Argentina; University of New Mexico, United States of America

## Abstract

*Staphylococcus aureus* is a microorganism that causes serious diseases in the human being. This microorganism is able to escape the phagolysosomal pathway, increasing intracellular bacterial survival and killing the eukaryotic host cell to spread the infection. One of the key features of *S. aureus* infection is the production of a series of virulence factors, including secreted enzymes and toxins. We have shown that the pore-forming toxin α-hemolysin (Hla) is the *S. aureus*–secreted factor responsible for the activation of the autophagic pathway and that this response occurs through a PI3K/Beclin1-independent form. In the present report we demonstrate that cAMP has a key role in the regulation of this autophagic response. Our results indicate that cAMP is able to inhibit the autophagy induced by Hla and that PKA, the classical cAMP effector, does not participate in this regulation. We present evidence that EPAC and Rap2b, through calpain activation, are the proteins involved in the regulation of Hla-induced autophagy. Similar results were obtained in cells infected with different *S. aureus* strains. Interestingly, in this report we show, for the first time to our knowledge, that both EPAC and Rap2b are recruited to the *S. aureus*–containing phagosome. We believe that our findings have important implications in understanding innate immune processes involved in intracellular pathogen invasion of the host cell.

## Author Summary

*Staphylococcus aureus* is a microorganism that causes serious infectious diseases such as pneumonia, endocarditis, osteomyelitis, and wound infections. This pathogen can infect various types of non-professional phagocytic cells and after internalization is able to escape the phagolysosomal compartment towards the cytoplasm, where it actively replicates. Subsequently, the eukaryotic host cell is killed to spread the infection. Besides the clinical importance of this microorganism, the molecular mechanisms of *S. aureus* infection are not completely understood. *S. aureus* induces an autophagic response in infected cells, which is beneficial for bacterial replication and cell killing. We have previously shown that Hla is responsible for this autophagy activation. We found that the Hla-induced autophagic response occurs by a “non-canonical" pathway independent of PI3K/Beclin1 complex but dependent on Atg5. Here we show that cAMP has a key role in the regulation of Hla-induced autophagic response. cAMP, through EPAC/Rap2b and via calpain activation, inhibits *S. aureus*–induced autophagy. Additionally, we show that EPAC and Rap2b are recruited to the *S. aureus*–containing phagosome. Our study contributes to the understanding of the molecular mechanisms used by *S. aureus* to survive, a key step in *Staphylococcal* pathogenicity.

## Introduction

Autophagy is a cellular process in response to stress, which is activated when cells are subjected to nutrient limitation, high temperatures, oxidative stress, accumulation of damaged organelles, or infection with certain pathogens [1]. When autophagy is activated, various cellular constituents, including long-lived proteins, cytoplasmic organelles, and some microorganisms, are encapsulated by the phagophore, a growing cistern that finally closes generating the autophagosome lined by two membranes. These vesicles intersect with the endosomal compartment, generating the amphisome, which finally fuses with lysosomes to become autolysosomes, where sequestered cellular components are digested and essential molecules are recycled back to the cytoplasm [2]. Genetic studies in yeast have led to the discovery of several Atg (autophagy related) genes, many of which have mammalian orthologs [3]. Atg12-Atg5 and the Atg8 systems are key components of the autophagic pathway. Atg5 interacts covalently with Atg12 and noncovalently with the multimeric protein Atg16. The microtubule-associated protein 1 light chain 3 (MAP1-LC3/Atg8/LC3) is cleaved at its C terminus by Atg4 to form LC3-I, which is covalently conjugated to phosphatidylethanolamine to generate LC3-II. LC3-II is formed where the Atg12-Atg5-Atg16 complex is localized and it remains associated with autophagosomes, even with mature autophagosomes/autolysosomes although at a lesser degree [4], [5].

Two main mechanisms involved in the regulation of the classical autophagy pathway have been described. One of them involves the serine/threonine kinase, mammalian target of rapamycin (mTOR), which inhibits autophagy and functions as a sensor for cellular energy and amino acid levels [3], [6]. The other one is through phosphatidylinositol-3-kinase (PI3K) Class III, which plays an important role in the activation of the autophagic pathway, acting as a positive regulator. Class III PI3K and its human ortholog hVps34 interact with Beclin 1 and p150 myristoylated kinase, activating some of the Atg proteins involved in the autophagic pathway [7]. More recently, a new kind of autophagic pathway independent of mTOR and rapamycin has been revealed [8]. Rubinsztein and coworkers demonstrated that autophagy can be induced by lowering intracellular inositol or inositol 1,4,5-trisphosphate (IP3) levels, in a mTOR-independent form [8], [9]. Consistently, Kroemer and collaborators have shown that genetic knockdown or pharmacological inhibition of the IP3 receptor (IP3R) induces autophagy [10]. Interestingly, it has been recently shown that IP3R represses autophagy through Bcl-2-mediated sequestration of Beclin 1 [11], thus linking IP3R with initial steps of the autophagic pathway.

Cumulative evidence indicates that autophagy is involved in the defense against several pathogen microorganisms [1], [12], [13]. Upon autophagy induction, intracellular bacteria such as *Streptococcus pyogenes*, *Mycobacterium tuberculosis*, and *Salmonella* are sequestrated within autophagosomes which then fuse with lysosomes to eliminate the intruder [13]. However, some pathogens like *Coxiella burnetti* and *Legionella pneumophila* benefit from autophagy and generate a replicative niche with autophagic features where the bacteria can actively replicate [12]. Other bacteria like *Shigella flexneri* and *Listeria monocytogenes* can escape from the phagosomes into the cytoplasm, where they multiply and generate actin tails to disseminate from the host cell to neighboring cells [12].

*Staphylococcus aureus* is a microorganism that causes serious diseases in humans. *S. aureus* has been classically considered an extracellular pathogen, but numerous studies have now shown that *S. aureus* can infect various types of non-professional phagocytic cells such as keratinocytes, fibroblasts, endothelial, and epithelial cells, leading to host cell death [14]–[17] *S. aureus* is able to escape the phagolysosomal pathway and, in this way, increase the intracellular bacterial survival and killing of the eukaryotic host cell, spreading the infection [18]. A previous study has shown that after the infection, *S. aureus* localizes to autophagosomes and inhibits its maturation and fusion with lysosomes [18]. Interestingly, *S. aureus* was not able to replicate in autophagy-deficient *Atg5*−/− mouse embryonic fibroblasts, indicating that *S. aureus* requires autophagy activation for replication, subsequent escape from autophagosomes into the cytoplasm, and *S. aureus*–induced host cell death [18].

We have previously demonstrated that α-hemolysin (Hla), which is a pore-forming toxin secreted by *S. aureus* [19], [20], is the factor responsible for the autophagic response induced by this pathogen in the host cell [21]. In addition, we have shown that this autophagic response induced by Hla occurs via a “non canonical" pathway, which is independent of Beclin1 and PI3K, but requires Atg5 [21]. Also, we have demonstrated that the LC3-labeled vesicles generated by the toxin are non-acidic and non-degradative compartments, indicating that somehow the toxin impedes the maturation of these autophagic structures [21].

In the present study, we have analyzed the signaling mechanisms that regulate the Hla-induced autophagy. We have identified some of the molecular components involved in this pathway and determined their participation in the regulation of the autophagic response generated by the toxin.

## Results

### cAMP Inhibits the Autophagic Response Induced by α-Hemolysin via a PKA-Independent Pathway

**Figure 1:**
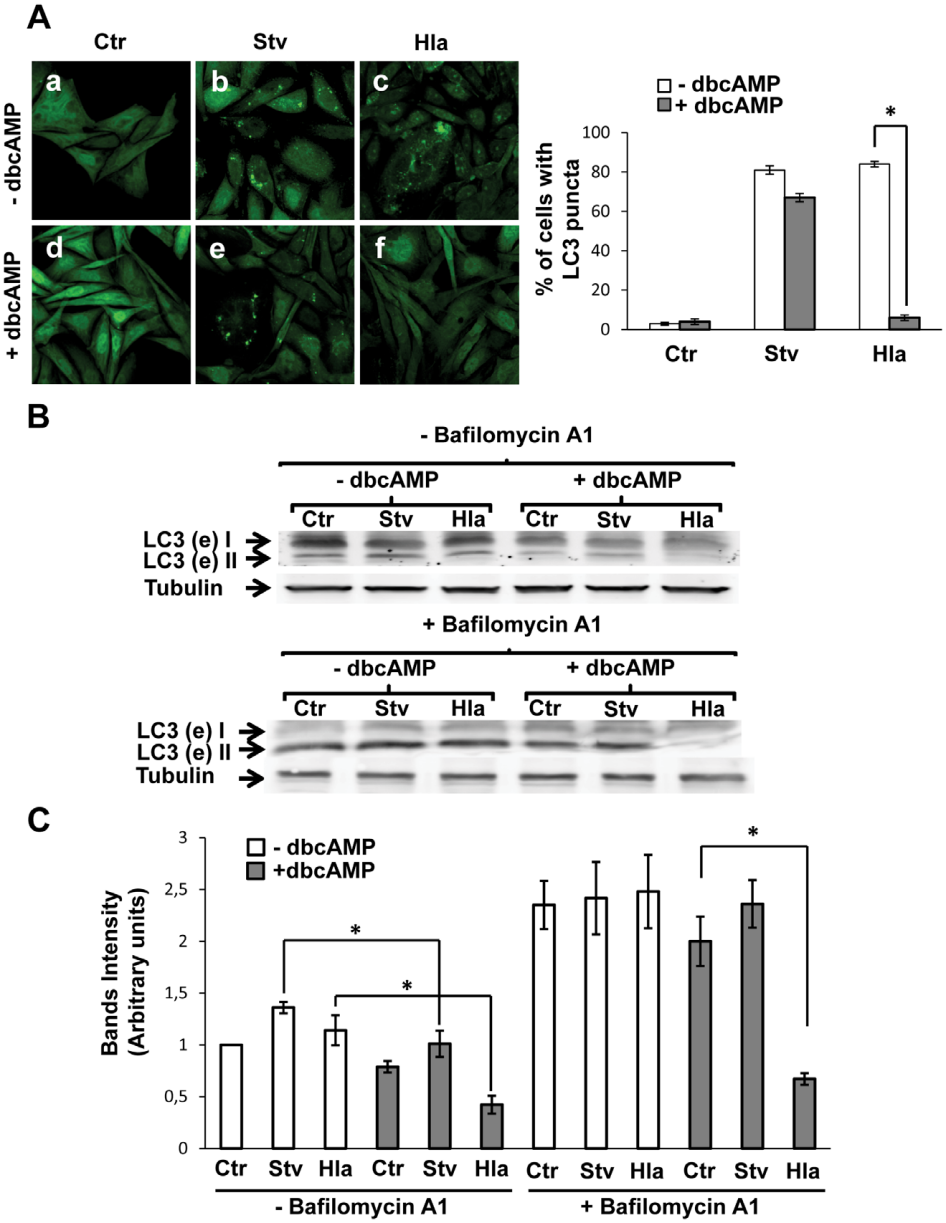
cAMP inhibits Hla-induced autophagy. (A) GFP-LC3 CHO cells were preincubated with 1 mM dybutiryl cAMP (+dbcAMP, panels d, e, and f) for 30 min and then were treated for 4 h with 10 µg/ml of α-hemolysin (Hla) or subjected to starvation conditions (Stv) in the continuous presence of dbcAMP. Cells without any treatment were used as control (−dbcAMP, panels a, b, and c). Cells were immediately analyzed by confocal microscopy. A quantification of the percentage of cells presenting LC3 puncta (i.e., stimulated cells) upon incubation with the different conditions is shown in the right panel (*n* = 100 cells/condition, * *p*<0.05 paired Student's t-test). These data are representative of three independent experiments. (B) GFP-LC3 CHO cells were incubated with complete medium in the absence (−dbcAMP) or presence of dbcAMP (+dbcAMP) and treated for 4 h with 10 µg/ml of α-hemolysin (Hla) or subjected to starvation conditions (Stv) with (lower panel) or without (upper panel) bafilomycin A1 to block lysosomal degradation. Afterwards, cells were lysed with sample buffer and the samples were subjected to Western blot analysis using a rabbit anti-LC3 and the corresponding HRP-labeled secondary antibody, and subsequently developed with an enhanced chemiluminescence detection kit. These data are representative of three independent experiments. (C) The band intensities of two independent experiments were quantificated with the Adobe Photoshop program, and normalized against tubulin. * *p*<0.05 (paired Student's t-test).

We have previously shown that the autophagic response induced by the toxin α-hemolysin (Hla) was not suppressed by the classical autophagy inhibitors 3-mehtyladenine or wortmannin, suggesting that this process occurs independently of PI3Kinase activation [21]. Thus, we were interested in determining whether other pathways might be involved in the regulation of this “non-canonical" autophagic response. In a recent publication, it was shown that cAMP-dependent protein kinase A (PKA) activation by cAMP is able to inhibit the autophagy pathway through LC3 phosphorylation [22] (Figure S1A). In order to determine if this pathway regulates the autophagic activation induced by alpha-toxin, we analyzed this process in stable transfected CHO cells overexpressing GFP-LC3. The protein LC3 is an autophagic marker present in eukaryotic cells as a soluble form (LC3-I) and a membrane-associated form (LC3-II). When autophagy is activated, LC3-I is conjugated to phosphatidylethanolamine to generate LC3-II, which localizes to autophagosomal membranes [5]. CHO cells overexpressing GFP-LC3 were incubated in complete medium, with or without the toxin or subjected to starvation conditions, in the absence or presence of dbcAMP, a permeable cyclic AMP (cAMP) analog. As shown in Figure 1A, cAMP caused a marked inhibition in the autophagic response induced by the toxin, as indicated by a decrease in LC3-positive vesicles (Panel f). In contrast, and to our surprise, just a little decrease in starvation-induced autophagy was observed (Panel e). The quantification of the percentage of cells presenting LC3 puncta upon incubation in the different conditions is shown in the right panel.

Next, we performed a Western blot assay to analyze the processing of LC3. CHO GFP-LC3 cells were incubated in complete medium, with or without the toxin, or subjected to starvation, with or without dbcAMP and in the presence (Figure 1B, lower panel) or the absence (Figure 1B, upper panel) of bafilomycin A1, an inhibitor of the H^+^ ATPase pump and autophagosome/lysosome fusion. In agreement with the results mentioned above, a decreased level of lipidated LC3 protein was detected in cells incubated with Hla in the presence of dbcAMP, even with bafilomycin A1 (Figure 1B). A quantification of the intensity of the bands is shown in Figure 1C. To corroborate these results, endogenous LC3 was also detected after the different conditions and similar results were obtained (Figure S2A).

**Figure 2:**
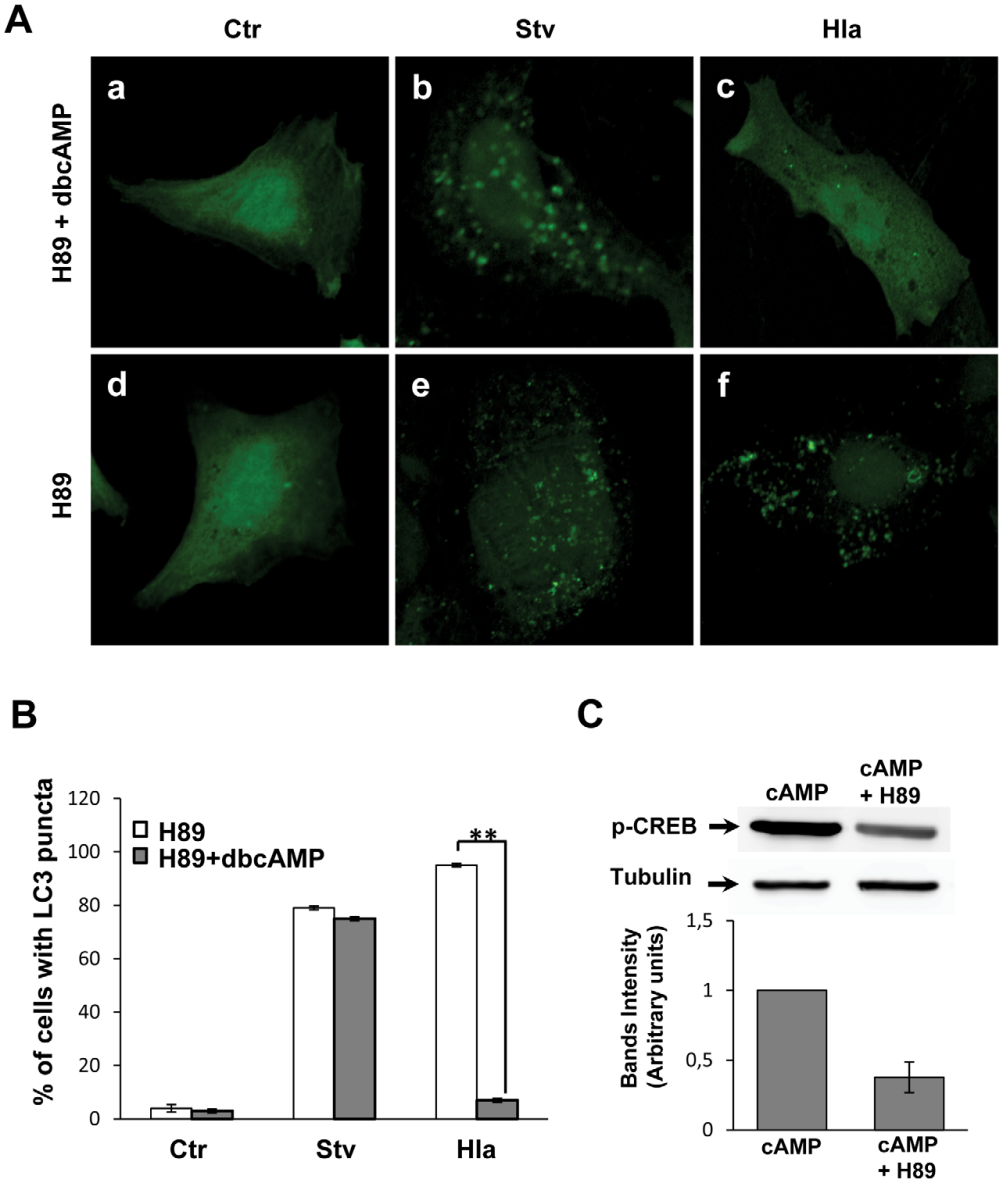
PKA inhibition does not affect the autophagic response induced by Hla. (A) GFP-LC3 CHO cells were preincubated for 30 min with 10 µM H89, a PKA inhibitor, in the presence (panels a, b, and c) or absence (panels d, e, and f) of 1 mM dbcAMP. Subsequently, they were incubated for 2 h in starvation medium (Stv, panels b and e) or treated for 4 h with 10 µg/ml of α-hemolysin (Hla, panels c and f) in full nutrient media. Cells without any treatment were used as control (Ctr, panels a and d). Cells were finally analyzed by confocal microscopy. Images are representative of three independent experiments. (B) Quantification of the percentage of cells presenting LC3 puncta (i.e., stimulated cells) upon incubation with the different conditions. ** *p*<0.01 (paired Student's t-test, *n* = 100 cells/condition). These data are representative of two independent experiments. (C) GFP-LC3 CHO cells were preincubated for 30 min in the presence or absence of 10 µM H89, and then they were incubated for 2 h with 1 mM dbcAMP to verify the activity of H89. Afterwards, cells were lysed with sample buffer and the samples were subjected to Western blot analysis using a rabbit anti-phosphoCREB and the corresponding HRP-labeled secondary antibody, and subsequently developed with an enhanced chemiluminescence detection kit. The band intensities were quantified with the Adobe Photoshop program (lower panel). These data are representative of two independent experiments.

In order to address whether PKA participates in cAMP-dependent inhibition of the toxin response, we incubated the cells with H89, a PKA inhibitor, in the presence or absence of dbcAMP (Figure 2A). As shown in Figure 2A, H89 was unable to revert the autophagy inhibition induced by dbcAMP in cells treated with the toxin (Panel c). In addition, we overexpressed a dominant-negative PKA regulatory subunit mutated in both sites A and B of the cAMP-binding domain [23]. This dominant negative mutant of the regulatory subunit of PKA cannot be activated by cAMP. Similar to the results obtained with H89, overexpression of this PKA inactive mutant was unable to antagonize the inhibitory effect of dbcAMP in the autophagic response induced by alpha toxin (data not shown). These results indicate that PKA is not participating in the cAMP-dependent inhibition of Hla-induced autophagy. In addition, H89 alone did not affect the autophagic response in either conditions (Figure 2A, Panels e and f). The quantification of the percentage of cells presenting LC3 puncta upon treatment with the different conditions is depicted in Figure 2B. To verify the activity of H89, we analyzed the phosphorylation of CREB, which is a PKA substrate, in cells stimulated with cAMP in the presence or absence of H89. As shown in Figure 2C, H89 decreased the phosphorylation of CREB, confirming that the PKA inhibitor is active in our system. These results indicate that cAMP is acting via a PKA-independent pathway.

### EPAC and Rap2b Negatively Regulates the Autophagy Induced by α-Hemolysin

**Figure 3:**
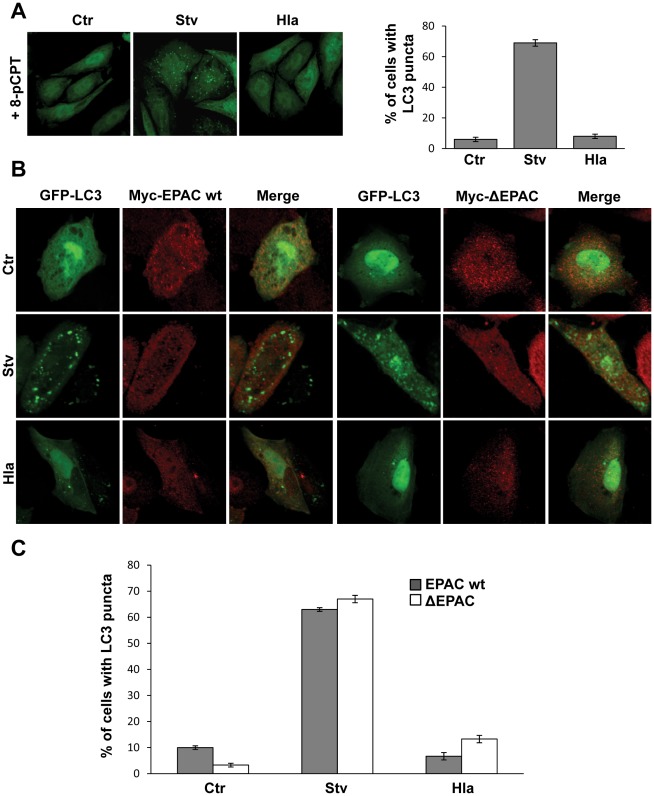
EPAC activation inhibits the Hla-dependent autophagy. (A) GFP-LC3 CHO cells were preincubated with 10 µM 8-pCPT-2′-O-Me-cAMP (8-pCPT) for 30 min and then they were treated for 4 h with 10 µg/ml of α-hemolysin (Hla) or subjected to starvation conditions (Stv). Cells without any treatment were used as control. Cells were immediately analyzed by confocal microscopy. Quantification of the percentage of cells presenting LC3 puncta upon incubation under the different conditions is shown in the right panel (*n* = 50 cells/condition). These data are representative of two independent experiments. (B) CHO cells were cotransfected with GFP-LC3 and myc-EPAC wt (left panel) or myc-ΔEPAC (right panel). Twenty-four hours after transfection they were incubated for 2 h in starvation medium (Stv) or treated for 4 h with 10 µg/ml of α-hemolysin (Hla). Cells without any treatment were used as control (Ctr). Cells were analyzed by confocal microscopy. Images are representative of two independent experiments. (C) Quantification of the percentage of cells presenting LC3 puncta upon incubation under the different conditions (*n* = 20 cells/condition). These data are representative of two independent experiments.

cAMP has traditionally been thought to act exclusively through PKA, but at present, cAMP is also known to directly regulate ion channels and the ubiquitous protein EPAC (exchange protein activated by cAMP), a cAMP-regulated effector that is a guanine nucleotide exchange factor (GEF) for the low molecular weight GTPase, Rap [24] (Figure S1A). 8-pCPT-2′-O-Me-cAMP (8-pCPT-cAMP) is a cAMP analog that specifically activates EPAC. Recently published studies demonstrate that 8-pCPT-cAMP is a useful tool to assess atypical actions of cAMP that are PKA-independent [25]. Thus, we next assessed the effect of 8-pCPT-cAMP on the autophagic response induced either by the toxin or by starvation. Similar to dbcAMP, 8-pCPT-cAMP was able to abolish the autophagic response upon α-hemolysin-treatment but did not substantially affect starvation-induced autophagy (Figure 3A). Thus, cAMP-induced EPAC activation seems to be sufficient to inhibit the autophagic response generated by Hla.

To ascertain the participation of the Rap exchange factor EPAC in the Hla-induced autophagic pathway, CHO cells were cotransfected with GFP-LC3 and Myc-EPAC wt or the Myc-ΔEPAC mutant, a constitutively active GEF mutant that maximally activates Rap even in the absence of cAMP stimulation [26]. Subsequently, the cells were incubated in complete medium (with or without 10 µg/ml of α-hemolysin) or under starvation conditions, a physiological inducer of autophagy. As shown in Figure 3B, overexpression of either EPAC wt (left panels) or its active mutant (ΔEPAC, right panels) was able to inhibit the Hla-induced autophagic response, but had no inhibitory effect in the autophagy induced by starvation. The quantification of the percentage of cells presenting LC3 puncta upon incubation in the different conditions is shown in Figure 3C. As control, CHO cells were cotransfected with GFP-LC3 and RFP-vector and treated as described above. As expected, both autophagy inducers (i.e., starvation and rapamycin), as well as treatment with the toxin, caused the typical LC3 punctate distribution (Figure S3A and S3B). Thus, taken together, these results indicate that EPAC regulates the autophagy induced by the toxin and when activated is able to inhibit this autophagic response.

**Figure 4:**
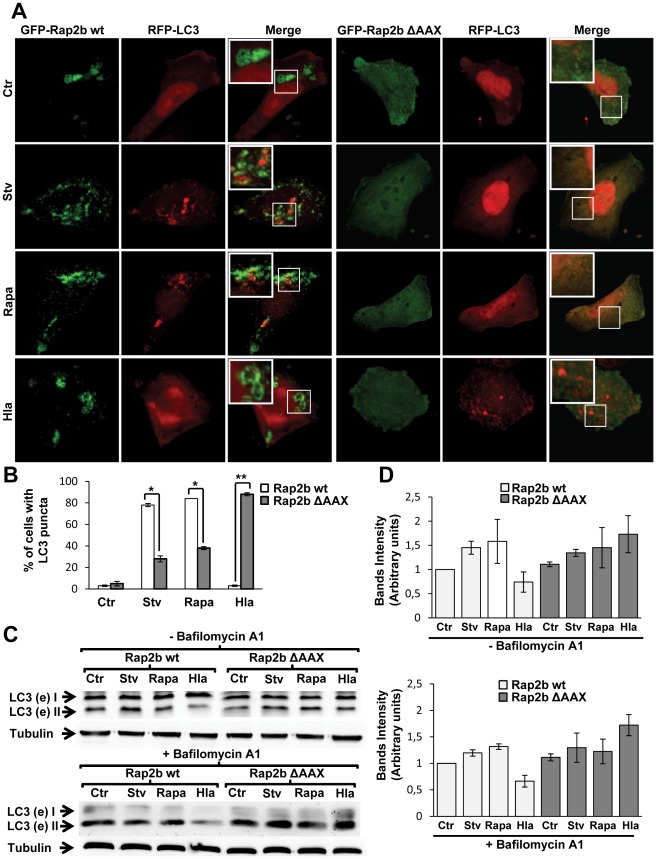
Rap2b negatively regulates the autophagic response induced by the toxin. (A) CHO cells were cotransfected with RFP-LC3 and GFP-Rap2b wt (left panel) or GFP-Rap2b ΔAAX (right panel). Twenty-four hours after transfection, they were incubated for 2 h in starvation medium (Stv), with 50 ng/µl of rapamycin (Rapa) in full nutrient media, or treated for 4 h with 10 µg/ml of α-hemolysin (Hla). Cells without any treatment were used as control (Ctr). Cells were analyzed by confocal microscopy. Images are representative of three independent experiments. (B) Quantification of the percentage of cells presenting LC3 puncta (i.e., stimulated cells) upon incubation with the different conditions is shown. * *p*<0.05, ** *p*<0.01 (paired Student's t-test, n = 50 cells/condition). These data are representative of three independent experiments. (C) CHO cells were transfected with GFP-Rap2b wt or GFP-Rap2b ΔAAX and incubated with complete medium without (Ctr) or with 10 µg/ml of α-hemolysin (Hla) for 4 h, with 50 ng/µl of rapamycin (Rapa) or subjected to starvation conditions (Stv) for 2 h in the absence or presence of bafilomycin A1. Afterwards, cells were lysed with sample buffer and the samples were subjected to Western blot analysis using a rabbit anti-LC3 and the corresponding HRP-labeled secondary antibody, and subsequently developed with an enhanced chemiluminescence detection kit. These data are representative of two independent experiments. (D) Quantification of the Western blot bands intensities of two independent experiments with the Adobe Photoshop program.

In order to corroborate whether the EPAC pathway is responsible for the regulation of autophagy activation induced by Hla, we analyzed a downstream component of this pathway, the small GTPase Rap2b. For this purpose, CHO cells were cotransfected with RFP-LC3 and GFP-Rap2b wt or the GFP-Rap2b ΔAAX mutant, which cannot be lipidated because it has a deletion in its C-terminal motif, losing both membrane localization and activity. It has been shown that deletion of the CAAX motif abolishes plasma membrane localization and compromises the biological function of many Ras-related GTPases [27]. The transfected cells were incubated in complete medium (in the presence or the absence of 10 µg/ml of α-hemolysin), under starvation conditions, or with 50 ng/µl of rapamycin, a pharmacological inducer of autophagy (Figure 4A). As control, CHO cells were cotransfected with RFP-LC3 and GFP-vector and treated as described above. As expected, both autophagy inducers (i.e., starvation and rapamycin), as well as treatment with the toxin, caused the typical LC3 punctate distribution (Figure S3C and S3D). As shown in Figure 4A, overexpression of Rap2b wt was able to inhibit the autophagic response induced by the toxin, but had no effect in the autophagy activated by starvation or rapamycin. In contrast, overexpression of the mutant Rap2b ΔAAX did not affect toxin-induced autophagy, indicating that Rap2b participates in the pathway that regulates the autophagy induced by α-hemolysin preventing this autophagic response. Of note, overexpression of Rap2b ΔAAX decreased the autophagy induced by starvation and rapamycin, suggesting that Rap2b might be a common link between both the “non-canonical" autophagy pathway induced by the toxin and the classical autophagic pathway induced by starvation. The quantification of the percentage of cells presenting LC3 puncta subjected to different treatments is shown in Figure 4B. To corroborate these results, we performed a Western blot assay to analyze the processing of LC3. CHO cells were transfected with Rap2b wt or Rap2b ΔAAX and incubated in complete medium (with or without the toxin) with rapamycin or subjected to starvation conditions in the presence or absence of bafilomycin A1. In agreement with the results mentioned above, a decreased level of lipidated LC3 protein was detected in cells overexpressing Rap2b wt and incubated with Hla, whereas no effect was observed in cells overexpressing the inactive mutant Rap2b ΔAAX (Figure 4C). A quantification of the bands intensities is shown in Figure 4D.

**Figure 5:**
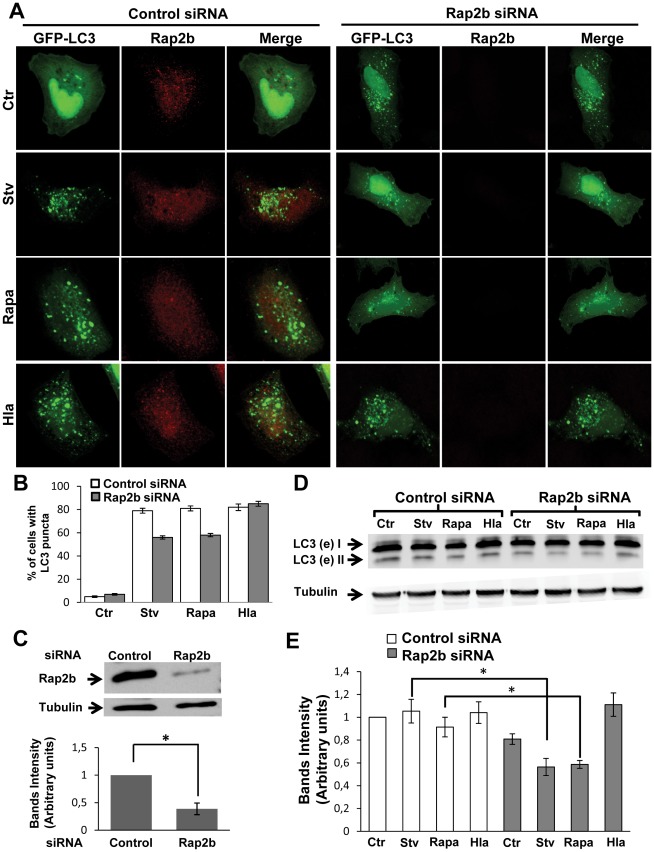
Rap2b knockdown does not affect Hla-induced autophagy. (A) HeLa cells were cotransfected with Rap2b siRNA and GFP-LC3 (right panel) or irrelevant siRNA and GFP-LC3 (left panel). Forty-eight hours after transfection, they were incubated for 2 h in starvation medium (Stv), with 50 ng/µl of rapamycin (Rapa) or treated for 4 h with 10 µg/ml of α-hemolysin (Hla). Cells without any treatment were used as control (Ctr). Cells were analyzed by confocal microscopy. Images are representative of two independent experiments. (B) Quantification of the percentage of cells presenting LC3 puncta upon incubation under the different conditions. Data correspond to two independent experiments (*n* = 50 cells/condition). (C) Upper panel: The knockdown of Rap2b was determined by Western blot as indicated in Materials and Methods. Lower panel: The band intensities of two independent experiments were quantified with the Adobe Photoshop program, and normalized against tubulin. * *p*<0.05 (paired Student's t-test). (D) HeLa cells were cotransfected with GFP-LC3 and Rap2b siRNA or an irrelevant siRNA and incubated for 4 h with complete medium in the absence (Ctr) or presence of 10 µg/ml of α-hemolysin (Hla), with 50 ng/µl of rapamycin (Rapa) or subjected to starvation conditions (Stv) for 2 h. Afterwards, cells were lysed with sample buffer and the samples were subjected to Western blot analysis using a rabbit anti-LC3 and the corresponding HRP-labeled secondary antibody, and subsequently developed with an enhanced chemiluminescence detection kit. These data are representative of two independent experiments. (E) The band intensities of two independent experiments were quantified with the Adobe Photoshop program, and normalized against tubulin. * *p*<0.05 (paired Student's t-test).

To determine whether depletion of Rap2b affects the starvation- or Hla-iduced autophagy, we used a Rap2b siRNA. HeLa cells were cotransfected with GFP-LC3 and Rap2b siRNA or with an irrelevant siRNA, and then they were incubated in complete medium (in the presence or the absence of 10 µg/ml of α-hemolysin) with rapamycin or under starvation conditions (Figure 5A). As shown in Figure 5A, knockdown of Rap2b was able to decrease the autophagic response induced by starvation and rapamycin, but did not affect the autophagy activated by the toxin. The quantification of the percentage of cells presenting LC3 puncta subjected to the different treatments is shown in Figure 5B. The effective knockdown of Rap2b was determined by Western blot as shown in Figure 5C. To corroborate these results, we performed a Western blot assay to analyze the processing of LC3. HeLa cells were transfected with Rap2b siRNA or irrelevant siRNA and subjected to the different treatments as indicated above. As shown in Figure 5D, a decreased level of lipidated LC3 protein was detected in cells transfected with Rap2b siRNA and incubated with rapamycin or under starvation conditions, but the levels were not affected in cells treated with the toxin. A quantification of the intensity of the bands is shown in Figure 5E.

**Figure 6:**
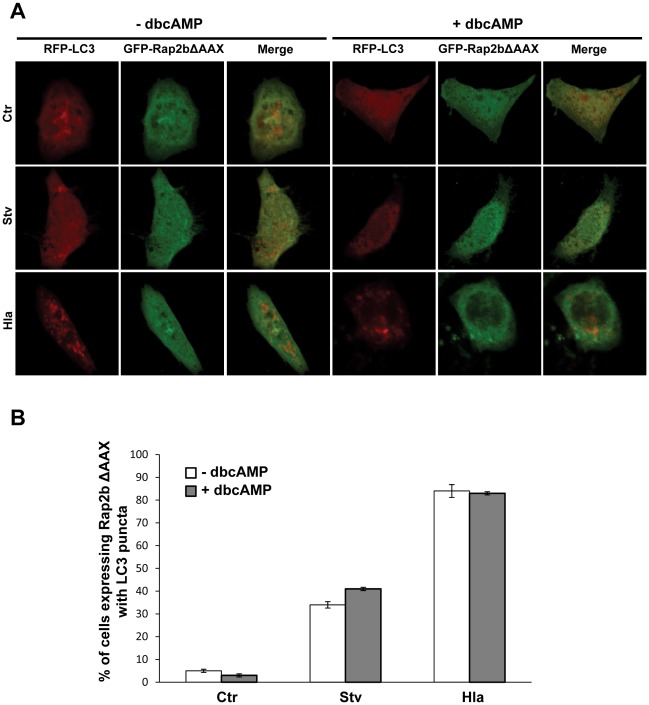
cAMP cannot inhibit the autophagy induced by the toxin in cells overexpressing the Rap2b negative mutant. (A) CHO cells were cotransfected with RFP-LC3 and GFP-Rap2b ΔAAX. Twenty-four hours after transfection they were incubated for 2 h in starvation medium (Stv) or treated for 4 h with 10 µg/ml of α-hemolysin (Hla) in the presence (right panels) or absence of 1 mM dbcAMP (left panels). Cells without any treatment were used as control (Ctr). Cells were analyzed by confocal microscopy. Images are representative of two independent experiments. (B) Quantification of the percentage of cells presenting LC3 puncta (i.e., stimulated cells) upon incubation with the different conditions. These data are representative of two independent experiments.

Next, we were interested in addressing whether cAMP was able to inhibit the Hla-induced autophagy even in cells overexpressing Rap2b ΔAAX. Thus, CHO cells were cotransfected with RFP-LC3 and GFP-Rap2b ΔAAX, and then they were treated with Hla or starvation medium in the presence or absence of cAMP (Figure 6A). Our results indicate that cAMP was unable to inhibit the autophagy induced by the toxin in cells overexpressing the Rap2b negative mutant, indicating that the inhibitory effect of cAMP requires a functional Rap2B (Figure 6A). CHO cells cotransfected with RFP-LC3 and GFP-vector and treated as above displayed the expected autophagic response induced by Hla or by starvation (data not shown). The quantification of the percentage of cells presenting LC3 puncta upon incubation in the different conditions is shown in Figure 6B.

Taken together, these results clearly indicate that Rap2b is a key participant in the regulation of autophagy induced by α-hemolysin, and it is likely that this small GTPase needs to be inactivated to allow this autophagic response.

### Inhibition of Calpains Allows Autophagy Activation Induced by the Toxin

**Figure 7:**
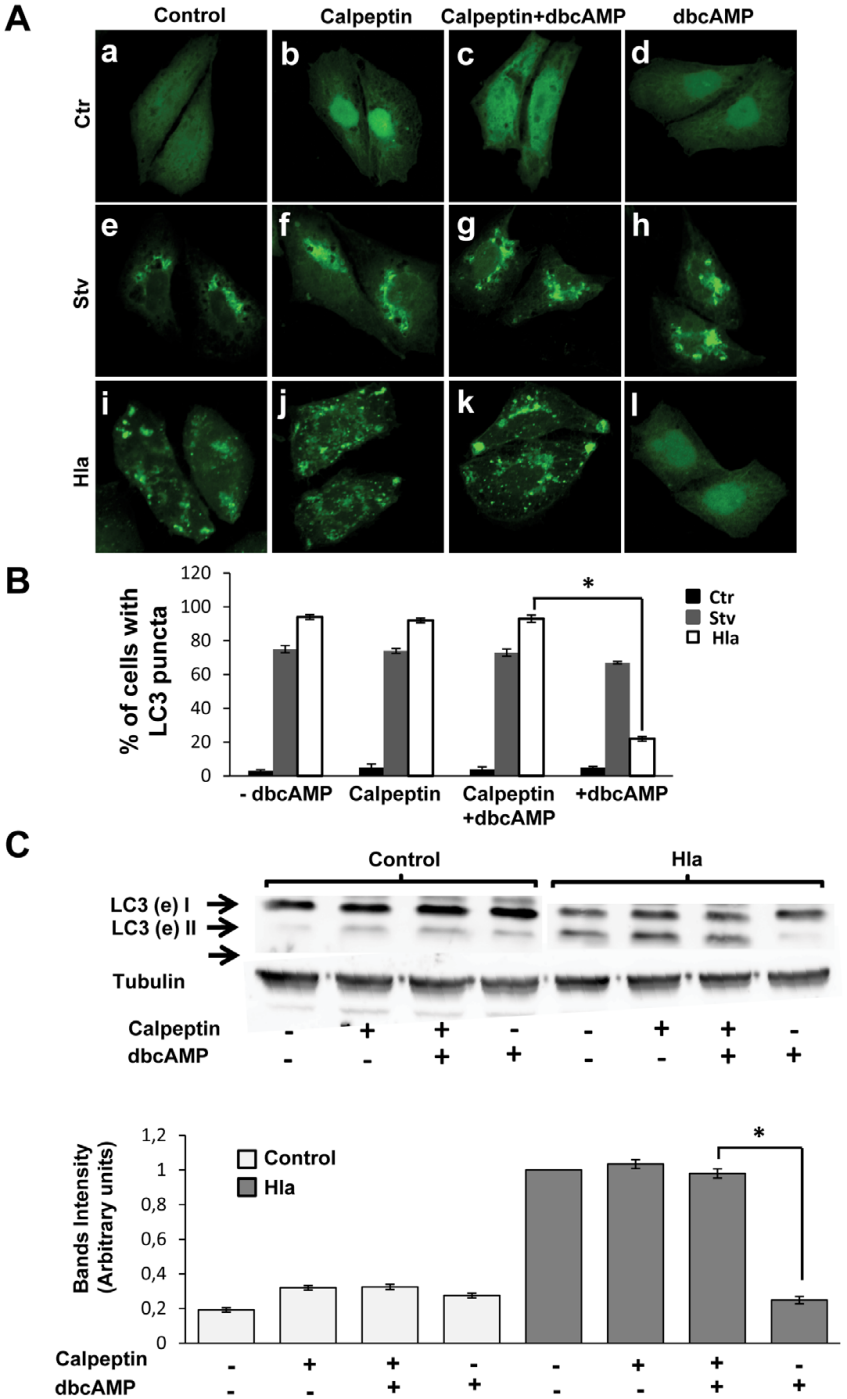
Calpain inactivation hampers the inhibitory effect of dbcAMP. (A) GFP-LC3 CHO cells were preincubated for 30 min with 10 µM of calpeptin (a calpain inhibitor, panels b, f, and j), with 1 mM dbcAMP (panels d, h and l), or with Calpeptin+dbcAMP (panels c, g, and k). Afterwards, they were incubated for 2 h in starvation medium (Stv, panels e, f, g, and h) or treated for 4 h with 10 µg/ml of α-hemolysin (Hla, panels i, j, k, and l). Cells without any treatment were used as control (Ctr, panels a, b, c and d). Cells were analyzed by confocal microscopy. Images are representative of two independent experiments. (B) Quantification of the percentage of cells presenting LC3 puncta (i.e., stimulated cells) upon incubation with the different conditions. * *p*<0.05 (paired Student's t-test, *n* = 100 cells/condition). These data are representative of two independent experiments. (C) Upper panel: CHO cells were preincubated for 30 min with 10 µM of calpeptin, with 1 mM dbcAMP, or with calpeptin+dbcAMP, and then they were incubated for 4 h in complete medium in the presence (Hla) or absence (Ctr) of α-hemolysin. Afterwards, cells were lysed with sample buffer and the samples were subjected to Western blot analysis using a rabbit anti-LC3 and the corresponding HRP-labeled secondary antibody. The bands were subsequently developed with an enhanced chemiluminescence detection kit. Lower panel: Quantification of the band intensities with the Adobe Photoshop program. * *p*<0.05 (paired Student's t-test). These data are representative of three independent experiments.

Rap2b is known to produce an increase of cytoplasmic calcium through a rise in IP3 [28]. Calpains are a family of cysteine-proteases that are activated by intracellular calcium. When calpains are activated, they are able to cleave Atg5, inhibiting autophagy in basal conditions [29]. In order to demonstrate the participation of calpains in this pathway we used calpeptin, a calpains inhibitor. CHO cells overexpressing GFP-LC3 were incubated with dbcAMP in the presence or the absence of calpeptin. Our results indicate that while cAMP alone was able to inhibit the Hla-induced autophagic response, calpeptin-preincubation prevented its inhibitory effect (Figure 7A, Panels k and l). These results suggest that during the autophagic response induced by Hla calpains are inhibited. The quantification of the percentage of cells presenting LC3 puncta upon treatment with the different conditions is shown in Figure 7B. In addition, the processing of LC3 was analyzed by Western blot. CHO cells were preincubated with dbcAMP, calpeptin, or calpeptin+dbcAMP, and then they were incubated in complete medium with or without the toxin. In agreement with the results mentioned above, calpeptin-preincubation prevented the inhibitory effect of dbcAMP in the Hla-induced autophagy, whereas a decreased level of lipidated LC3 protein was detected in cells preincubated with dbcAMP alone and treated with Hla (Figure 7C). A quantification of the bands' intensities is shown in Figure 7C (lower panel). Thus, these results suggest that the activation of calpains by cAMP leads to the inhibition of the Hla-induced autophagy. Therefore, calpains might act as negative regulators of this particular form of toxin-induced autophagic response.

### cAMP, EPAC, and Rap2b Negatively Regulate the Autophagic Response Induced upon Infection with *S. aureus*

**Figure 8:**
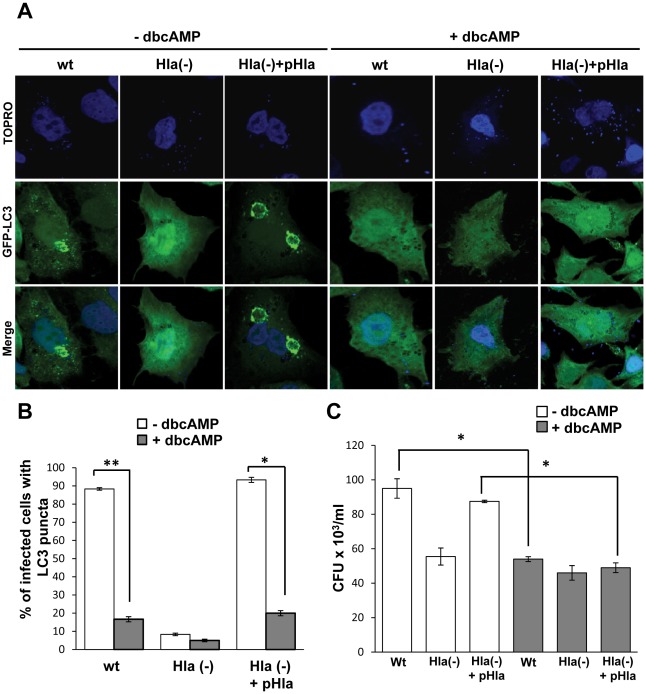
cAMP inhibits *S. aureus*–induced autophagy. (A) GFP-LC3 CHO cells were preincubated with 1 mM dybutiryl cAMP (+dbcAMP) for 30 min and then were infected for 4 h in the presence of db cAMP with the wt strain of *S. aureus* (wt), the mutant deficient for α-hemolysin (Hla−), or the Hla(−) mutant expressing an α-hemolysin plasmid (Hla(−) +pHla). Cells without any treatment were used as control (−dbcAMP). The nucleus and the bacteria were labeled with TOPRO as indicated in Materials and Methods, and immediately visualized by confocal microscopy. Images are representative of two independent experiments. (B) Quantification of the percentage of cells presenting LC3 puncta (i.e., stimulated cells) upon incubation in the different conditions. * *p*<0.05, ** *p*<0.01 (paired Student's t-test, *n* = 50 cells/condition). These data are representative of two independent experiments. (C) Quantification of the number of CFU/ml of CHO cells infected for 3 h with the wt strain of *S. aureus*, the mutant deficient for α-hemolysin (Hla−), or the Hla(−) mutant expressing an α-hemolysin plasmid in the absence or presence of cAMP. * *p*<0.05 (paired Student's t-test). These data are representative of two independent experiments.

We have previously shown that a population of internalized *S. aureus* recruits GFP-LC3 to their containing phagosomes and that this recruitment was dependent on the production of α-hemolysin [21]. To corroborate the participation of the above pathway in the regulation of autophagy induced by the toxin, we used different *S. aureus* strains: a wild-type strain (wt), a mutant deficient for α-hemolysin (Hla−), and the Hla(−) mutant complemented with an α-hemolysin plasmid (Hla(−)+pHla). GFP-LC3 CHO cells were preincubated with dbcAMP and infected for 4 h with the different *S. aureus* strains. Cells were then incubated with TOPRO, a DNA marker, to label the bacteria. As shown in Figure 8A, right panels, cAMP caused inhibition of autophagy induced either by *S. aureus* wt or by the complemented Hla(−) mutant. As control, autophagy activation by the wt and the complemented Hla(−) mutant strains in the absence of dbcAMP is also depicted (Figure 8A, left panels). The quantification of the percentage of infected cells presenting LC3 puncta upon treatment with or without dbcAMP is shown in Figure 8B.

We have previously shown that autophagy inhibition decreases bacterial replication [21], so next we determined whether cAMP affects intracellular bacterial grown. For this purpose, CHO cells were infected for 3 h with the wt strain of *S. aureus*, the mutant deficient for α-hemolysin (Hla−), or the Hla(−) mutant expressing an α-hemolysin plasmid. After the infection, cells were lysed and plated for colony forming units (CFU) quantification (Figure 8C). Interestingly, a marked decrease in bacterial replication was observed after treatment with cAMP, corroborating that autophagy is necessary for efficient bacterial replication and demonstrating that elevated levels of cAMP negatively affects *S. aureus* intracellular growth.

**Figure 9:**
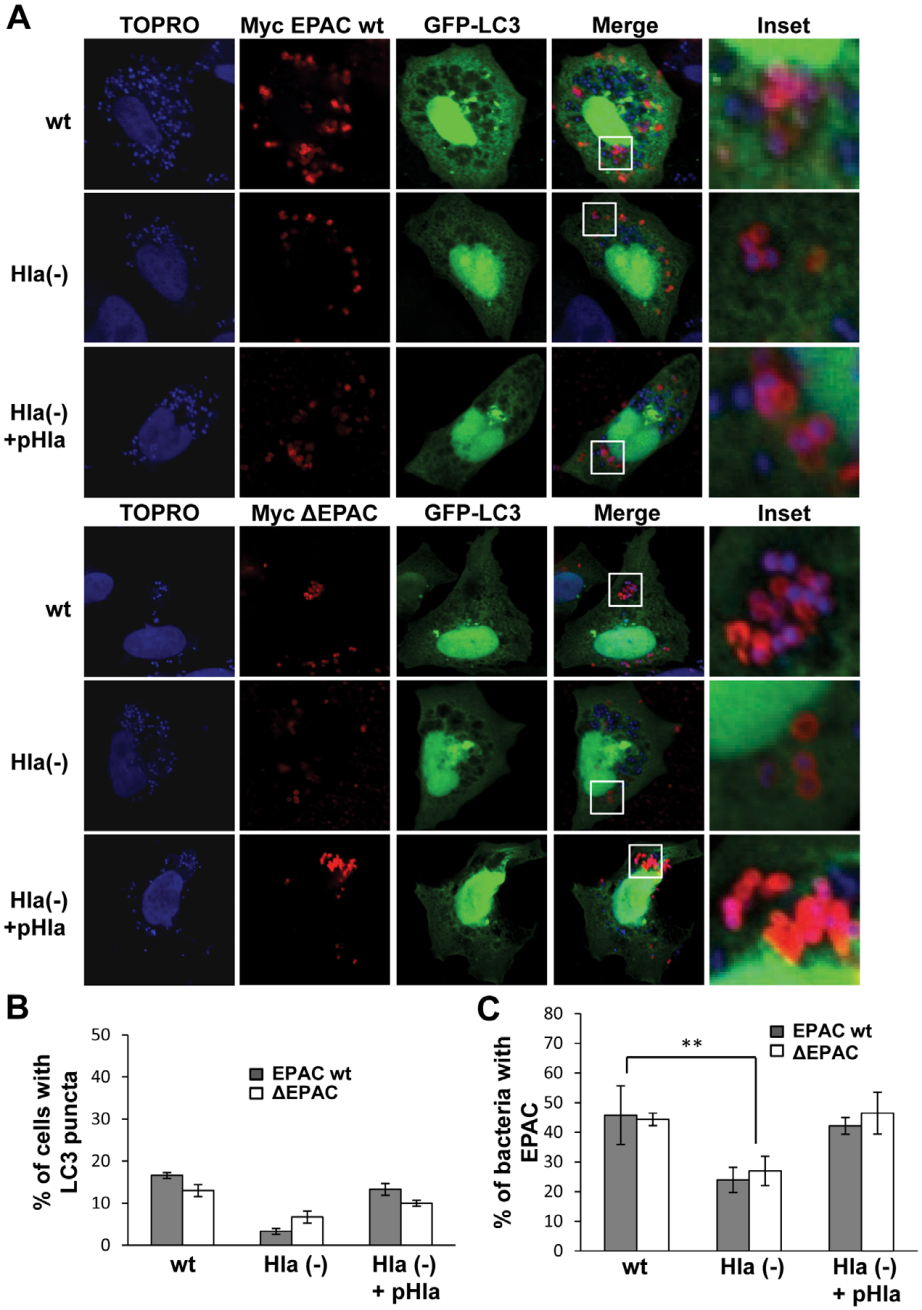
EPAC overexpression suppresses the autophagic response induced by *S. aureus*. CHO cells were cotransfected with GFP-LC3 and myc-EPAC wt (upper panel) or myc-ΔEPAC (lower panel). Twenty-four hours after transfection, they were infected for 4 h with the wt strain of *S. aureus* (wt), the mutant deficient for α-hemolysin (Hla−), or the Hla(−) mutant expressing an α-hemolysin plasmid (Hla(−)+pHla). The nucleus and the bacteria were labeled with TOPRO as indicated in Materials and Methods and immediately visualized by confocal microscopy. Images are representative of three independent experiments. (B) Quantification of the percentage of cells presenting LC3 puncta upon the infection. These data are representative of three independent experiments. (C) Quantification of the percentage of bacteria decorated with EPAC in cells infected with *S. aureus*. ** *p*<0.01 (paired Student's t-test, *n* = 20 cells/condition). These data are representative of three independent experiments.

**Figure 10:**
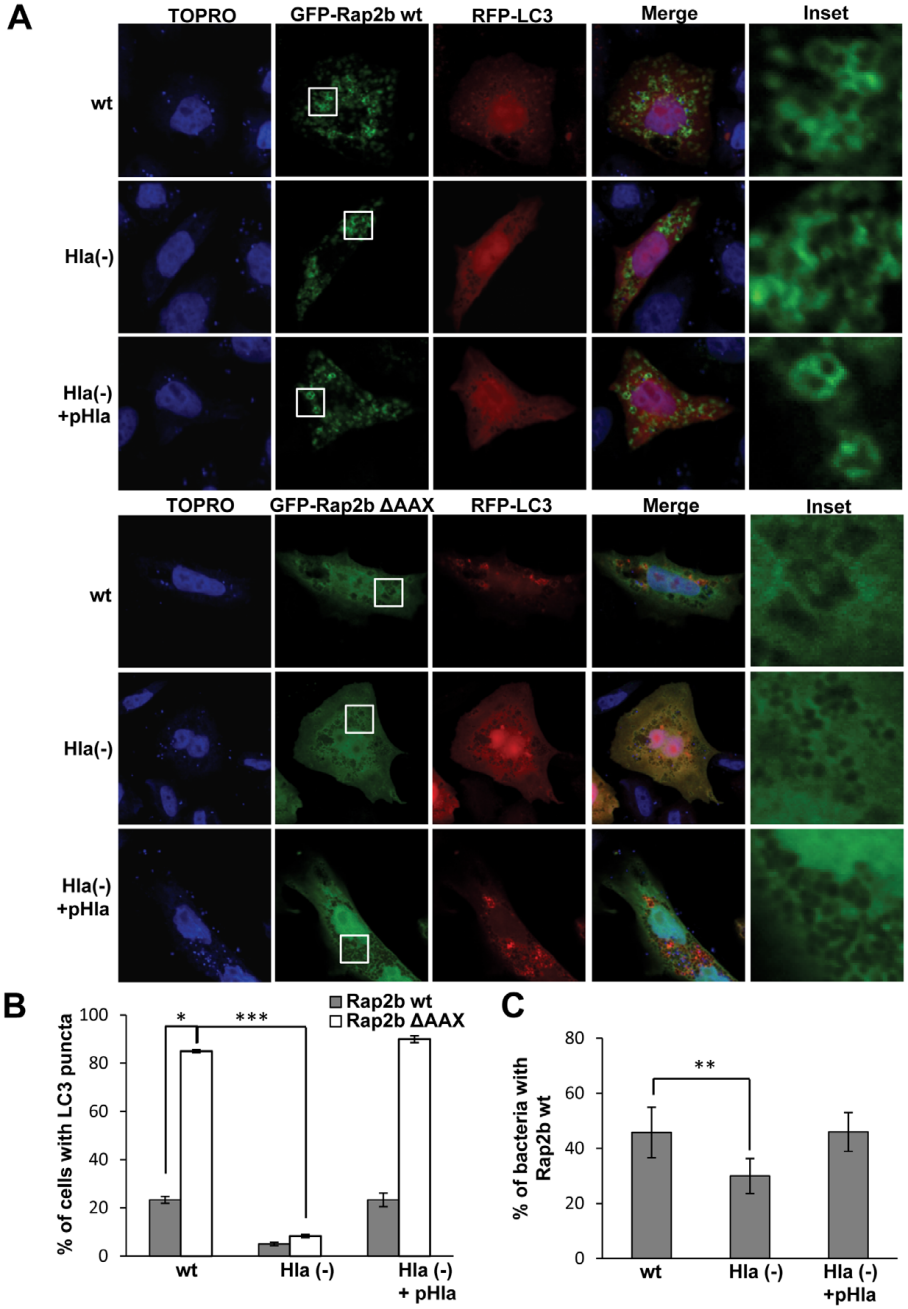
Rap2b inhibits the autophagy induced by *S. aureus*. (A) CHO cells were cotransfected with RFP-LC3 and GFP-Rap2b wt (upper panel) or GFP-Rap2b ΔAAX (lower panel). Twenty-four hours after transfection, they were infected for 4 h with the wt strain of *S. aureus* (wt), the mutant deficient for α-hemolysin (Hla−), or the Hla(−) mutant expressing an α-hemolysin plasmid (Hla(−)+pHla). The nucleus and the bacteria were labeled with TOPRO as indicated in Materials and Methods and immediately visualized by confocal microscopy. These data are representative of three independent experiments. (B) Quantification of the percentage of infected cells presenting LC3 puncta upon the infection. * *p*<0.05, *** *p*<0.001 (paired Student's t-test, *n* = 50 cells/condition). These data are representative of three independent experiments. (C) Quantification of the percentage of bacteria decorated with Rap2b wt upon the infection. ** *p*<0.01 (paired Student's t-test, *n* = 30 cells/condition). These data are representative of three independent experiments.

In order to corroborate the participation of EPAC and Rap2b in this autophagy regulation, CHO cells were cotransfected with GFP-LC3 and myc-EPAC wt or myc-ΔEPAC (Figure 9A); or with RFP-LC3 and GFP-Rap2b wt or GFP-Rap2b ΔAAX (Figure 10A). Cells were then incubated with TOPRO, to label the bacteria. As shown in Figure 9A, overexpression of EPAC wt (upper panels) or its active mutant ΔEPAC (lower panels) was sufficient to suppress the autophagic response induced by *S. aureus* wt and the complemented Hla(−) mutant (Hla(−)+pHla). The quantification of the percentage of infected cells overexpresing EPAC presenting LC3 puncta is shown in Figure 9B. As control, CHO cells were cotransfected with GFP-LC3 and RFP-vector and infected as described above. As expected, both the wt strain and the complemented Hla(−) mutant caused the typical LC3 recruitment (Figure S4).

Consistently, overexpression of Rap2b was also able to inhibit the autophagy induced by the bacteria (Figure 10A, upper panels). In contrast, overexpression of the negative mutant Rap2b ΔAAX had not effect in the autophagic response induced by *S. aureus* (Figure 10A, lower panels). The quantification of the percentage of infected cells overexpresing Rap2b presenting LC3 puncta is shown in Figure 10B. Strikingly, a remarkable recruitment of both EPAC and Rap2b to the bacteria-containing phagosome was observed (insets in Figures 9A and 10A). The quantification of the percentage of bacteria decorated with EPAC or Rap2b upon the infection is shown in Figure 9C and 10C. These data indicate that approximately 40%–50% of the bacteria-containing phagosomes showed association with either EPAC or Rap2B. The association of both proteins to the *S. aureus* phagosomal compartment was only partly decreased in phagosomes containing the Hla-deficient strain. For this reason, we determined the localization of endogenous EPAC or Rap2b. CHO cells were infected with the different *S. aureus* strains and EPAC or Rap2b were detected by indirect immunofluorescense (Figure S5). Interestingly, we observed that the Hla-deficient strain was unable to recruit either EPAC or Rap2b, suggesting that the partial recruitment of the overexpressed EPAC and Rap2b by the Hla (−) strain is likely due to the excess of molecules present in the transfected cells. Next, we analyzed the colocalization between LC3 and endogenous EPAC or Rap2b. For this purpose, CHO GFP-LC3 cells were infected as above and the proteins were detected by indirect immunofluorescense (Figure S6). Interestingly, neither EPAC nor Rap2b colocalized with LC3, suggesting that the population of bacteria that recruit EPAC/Rap2b does not recruit LC3 or that both proteins are differentially recruited on time.

Taken together, these results clearly confirm that the identified molecular components involved in the autophagic pathway induced by the purified toxin also participate in the autophagic response upon infection with *S. aureus*, negatively regulating the autophagic response exerted by this pathogen.

### Active Rap2b Are Downregulated to Allow the Autophagic Response Induced by Hla and upon Infection with *S. aureus*

**Figure 11:**
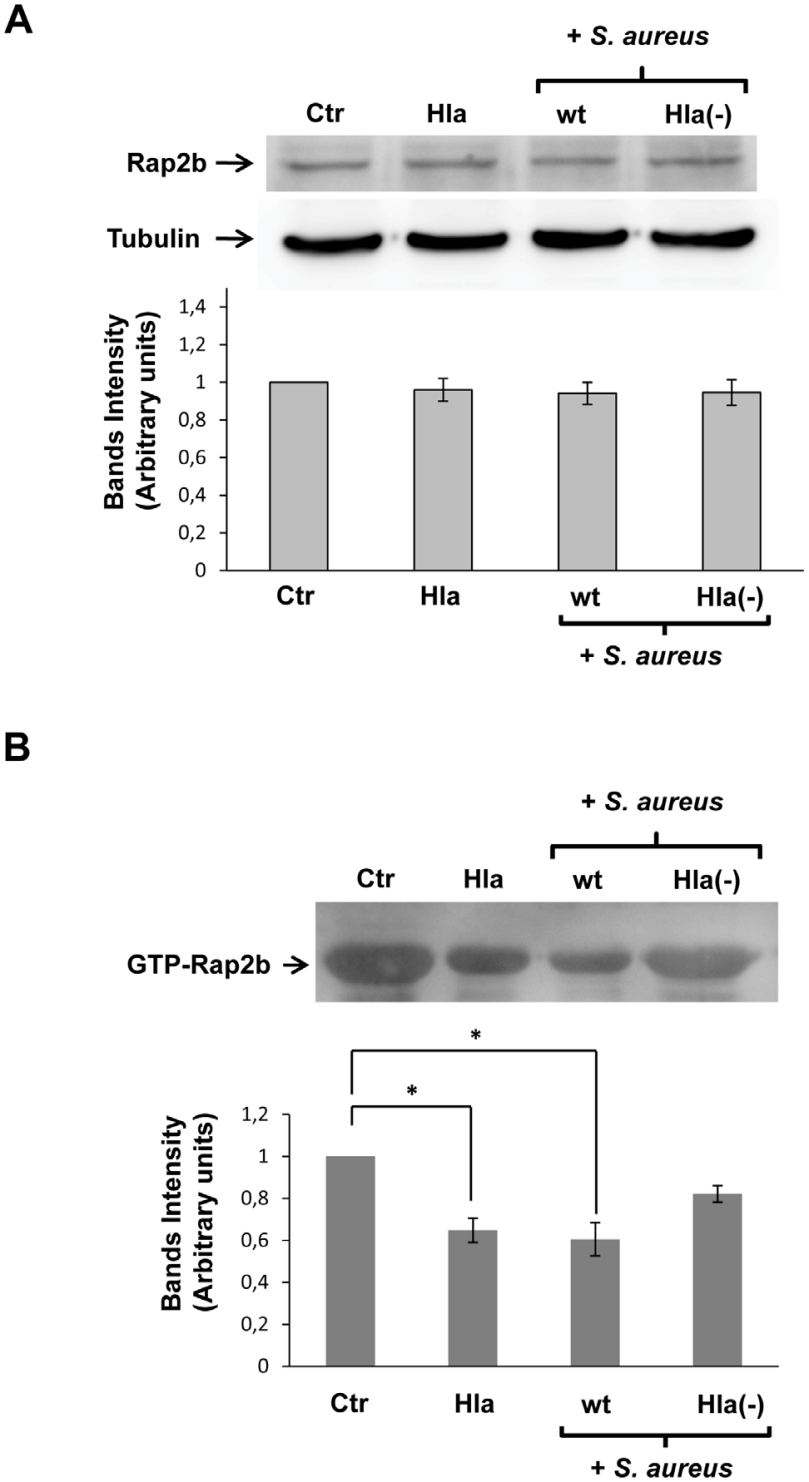
Active Rap2b is decreased to allow the autophagy response induced by Hla and *S. aureus*. (A) HeLa cells were incubated for 4 h with complete medium in the presence (Hla) or absence (Ctr) of 10 µg/ml α-hemolysin or they were infected for 4 h with the wt strain of *S. aureus* (wt) or the α-hemolysin deficient mutant (Hla−). Afterwards, cells were lysed with sample buffer and the samples were subjected to Western blot analysis using a rabbit anti-Rap2b and the corresponding HRP-labeled secondary antibody. The bands were subsequently developed with an enhanced chemiluminescence detection kit. A quantification of the bands intensities with the Adobe Photoshop program is shown in the lower panel. These data are representative of two independent experiments. (B) HeLa cells were incubated for 4 h with complete medium in the presence (Hla) or absence (Ctr) of 10 µg/ml α-hemolysin or they were infected for 4 h with the wt strain of *S. aureus* (wt) or the α-hemolysin deficient mutant (Hla−). Cells were disrupted and whole cell lysates were subjected to pull-down assays using GST-Ral-GDS-RBD-sepharose. The levels of GTP-bound Rap2b were determined as was described in Materials and Methods by Western blot analysis using a rabbit anti-Rap2b and the corresponding HRP-labeled secondary antibody. The bands were subsequently developed with an enhanced chemiluminescence detection kit. The band intensities were quantificated with the Adobe Photoshop program is shown in the lower panel. * *p*<0.05 (paired Student's t-test). These data are representative of two independent experiments.

The results shown above clearly indicate that cAMP and Rap2b are negative regulators of the autophagic activation induced by the toxin and by the infection with *S. aureus*. In order to determine how the bacteria and the toxin are able to regulate this autophagic pathway, we analyzed the levels of active Rap2b present in the cells. For this purpose, HeLa cells were incubated with the toxin or infected for 4 h with *S. aureus* wt strain or the mutant deficient in Hla. Then, the cells were lysed and GTP-bound Rap2b was pulled down with immobilized Ral-GDS-RBD, a cassette that is able to bind GTP-Rap proteins [30]. The amount of protein pulled down was determined by Western blot assay, using a polyclonal Rap2b antibody. As shown in Figure 11B, the levels of active Rap2b decreased in response to Hla treatment or when the cells were infected with *S. aureus* wt strain. However, no differences in the total amount of cellular Rap2b in the cells subjected to the different conditions were observed (Figure 11A). These results suggest that the toxin induces a decrease in the amount of active Rap2b, likely by decreasing the levels of intracellular cAMP, to allow autophagy induction, a response that favors pathogen intracellular survival as previously demonstrated.

A model indicating the two cAMP-dependent pathways involved in the control of autophagy and the effect of different molecules used in this study on the autophagic response induced by Hla and by *S. aureus* is shown in Figure S1B.

## Discussion

In previous studies we have demonstrated that *S. aureus* induces autophagy activation in infected cells [21]. We presented evidence indicating that *S. aureus* uses the α-hemolysin to activate the autophagic response, generating LC3-positive vesicles that are unable to mature, interrupting the autophagic flux [21]. These autophagic vesicles, which are non-acidic and non-degradative compartments, are likely used by the bacteria as a replicative niche, escaping then toward the cytoplasm to subsequently infect neighboring cells. We have also shown that the autophagic response induced by Hla does not occur by the classical pathway of autophagy activation. The toxin uses an alternative form to induce autophagy, which is independent of the PI3K/Beclin1 complex but dependent on the autophagic protein Atg5 [21].

In the present study, we have shown that cAMP plays a key role in the Hla-induced autophagic response. Indeed, our results indicate that this response is strongly suppressed by cAMP treatment. cAMP is a classical PKA activator that participates in several cellular process [31]. Recently, it has been demonstrated that PKA participates in autophagy regulation induced by the rapamycin-mediated inactivation of the TOR pathway [22]. In that report, Charleen Chu and coworkers have shown that, upon activation by cAMP, PKA is able to phosphorylate LC3. This phosphorylation in LC3 prevents autophagy activation, suggesting cAMP as a possible regulator of the autophagic pathway induced by rapamycin [22]. In addition, it has been also shown in the budding yeast *S. cerevisiae* that elevated levels of Ras/PKA activity prevented the autophagy activity induced by either nitrogen starvation or by the pharmacological inducer rapamycin [32]. It was proposed that this signaling pathway is controlling an activity required during the early stages of the autophagic pathway. However, in our work, we have demonstrated by employing the widely used PKA inhibitor H89 [33] (Figure 2), and by overexpressing a dominant negative mutant of the regulatory subunit of PKA [23] (data not shown), that PKA does not seem to participate in cAMP inhibition of the autophagic pathway induced by the toxin.

For many years, cAMP signaling was solely associated with PKA. However, novel cAMP sensors have come to light and they regulate many physiological processes either in concert with PKA or by themselves. It is known that cAMP is able to stimulate the cAMP-activated guanine exchange factor EPAC, which specifically turns on the monomeric G protein Rap [24], [34], [35]. EPAC proteins are known to control a range of diverse effectors and to regulate several pivotal processes. Here, we have shown that EPAC and its effector Rap2b participate in the regulation of Hla-induced autophagy. We have demonstrated that the direct activation of EPAC by cAMP or the overexpression of EPAC/Rap2b is sufficient to inhibit the autophagy response induced by the toxin (Figures 3, 4, and 6). Interestingly, Rubinsztein and collaborators [28] have shown that drugs that signal via the imidazoline type 1 receptor (I1R), such as clonidine, act by reducing cAMP levels. The compounds enhanced A53T α-synuclein clearance and decreased toxic protein aggregation by activating an m-TOR independent autophagy. In addition, these I1R agonists signal via cAMP/Epac/Rap2b/PLC. When activated by cAMP, EPAC in turn activates Rap2b, which, through PLCε and an increase in the cytoplasmic levels of IP3, induces exit of calcium from the endoplasmic reticulum. Rise in intracytosolic Ca2+ activates the calcium-dependent cysteine-protease calpains [36]. Indeed, it was shown that calpain inhibitors and siRNA knockdown of either calpain 1 or calpain 2 increased LC3-labeled autophagosomes [28]. Likewise, Junying Yuan and coworkers have shown that flurispirene, a compound that inhibits calcium flux, activates autophagy [29]. These authors have demonstrated that inactivation of calpain 1, which in turn is able to cleave Atg5, leads to activation of autophagy by increasing the levels of the Atg5-Atg12 complex required for LC3-lipidation. Consistently, with both reports, we have shown that the inhibition of calpains by the inhibitor calpeptin was sufficient to revert cAMP inhibition of the autophagy induced by Hla (Figure 7). Our results demonstrate, to our knowledge for the first time, that this signaling pathway participates in the regulation of the Hla-induced autophagic response and suggest that the toxin likely controls this pathway to allow autophagy activation, which is beneficial to the bacteria [18], [21]. Additionally, a role for Atg5 in apoptosis has also been demonstrated. Hans-Uwe Simon and collaborators have identified a truncated form of Atg5 of 24 kDa in human neutrophils and Jurkat cells that is generated following different stimuli [37]. They concluded that Atg5 is cleaved by calpain 1 and 2. They also showed that cells overexpressing Atg5 are more sensitive to cell death induced by different apoptotic stimuli and that the silencing of Atg5 reduces this cell death. Interestingly, this truncated Atg5 translocates from cytoplasm to mitochondria and causes cytochrome c release. The truncated form of Atg5 binds to Bcl-xl and may inactivate the Bcl-xl anti-apoptotic activity, promoting apoptotic cell death. These results clearly represent a link between autophagy and apoptosis through the calpain-mediated Atg5 cleavage [37]. Thus, it is tempting to speculate that *S. aureus* inhibits the calpain-mediated Atg5 cleavage to avoid apoptotic cell death and to favor autophagy activation, which is known to promote bacterial replication and bacterial survival [18].

To confirm the participation of this cAMP/EPAC/Rap2b pathway in the bacterial infection process, we used different *S. aureus* Hla positive and null strains. We have demonstrated that preincubation with cAMP was also able to inhibit the autophagy activation induced by *S. aureus*. Similar results were obtained when Rap2b wt and EPAC wt or its constitutively active mutant ΔEPAC were overexpressed. Interestingly, both EPAC and Rap2b were markedly recruited to the membrane of the bacteria-containing phagosome (Figure 9 and Figure 10). Aronoff and collaborators have demonstrated that following treatment of alveolar macrophages with prostaglandin E2, EPAC-1 changes its localization from tubular membranes to the nuclear envelope and late phagosomes [38]. Since it has been shown that 8-pCPT, via EPAC, inhibits H_2_O_2_ production and bacterial killing, we propose that EPAC is recruited to the *S. aureus* phagosomal membrane to suppress its microbicidal capacity, inhibiting the killing of this intraphagosomal pathogen [38], [39]. To the best of our knowledge, our studies are the first to demonstrate the recruitment of EPAC and Rap2b to a pathogen-containing compartment. Additionally, we have observed that those phagosomes that recruit EPAC are not labeled by LC3 (Figure S6). This is consistent with our model that EPAC (and Rap2b) act as an inhibitory molecule of the autophagic response induced by the Hla toxin. Further studies will be necessary to determine whether EPAC recruitment affects pathogen survival. We believe that our findings have important implications in understanding innate immune processes. Experiments are under way in our laboratory to determine how EPAC modulation controls the microbicidal capacity of different bacterial-containing phagosomes.

Additionally, our results suggest that *S. aureus* keeps autophagy under tight control by downregulating levels of active Rap2b (Figure 11), likely to maintain appropriate levels of the Atg5-Atg12 conjugate (Figure S2B). Since cAMP through EPAC activation is able to activate Rap2b, we determined the intracellular cAMP levels by RIA as described in Materials and Methods. We have observed a decrease in cAMP level in cells treated with the toxin or infected with *S. aureus* wt strain (data not shown). Inhibition of calpain activity as a result of reductions in intracellular Ca2+ might be part of the signal that leads to the activation of autophagy machinery by increasing the levels of a key autophagy signaling molecule such as Atg5. Indeed, we have previously shown that Atg5 is an absolute requirement for the toxin-activated autophagic response [21]. The evidence presented in this report indicates that the complex interplay between cAMP and Ca2+, known to be involved in the control of many cellular processes, may also expand to the regulation of a pathogen-induced autophagic response. We have previously shown that a high concentration of BAPTA-AM (30 µM), an intracellular calcium chelator, is able to avoid the Hla-induced autophagy [21]. This concentration of BAPTA-AM allows the compound to cross the plasma membrane and the membrane of some organelles, chelating not only cytosolic but also intravesicular calcium [40]. Interestingly, we have found that a lower concentration of BAPTA-AM (5 µM), which chelates only the cytosolic calcium [41], allows the autophagic response induced by the toxin (data not shown). It is known that a certain level of intracellular calcium is necessary for autophagosome formation [42], but we believe that an excess of cytosolic calcium leads to activation of the calpains proteases, which in turn could arrest the autophagic response induced by Hla. Thus, is likely that the intracellular calcium concentration is tightly regulated upon infection of *S. aureus*. Given the fact that *S. aureus* is a microorganism that causes serious diseases such as pneumonia, endocarditis, osteomyelitis, and wound infections, we believe that knowledge of the signal transduction mechanisms involved in the autophagy response and how these mechanisms enhance the intracellular survival of *S. aureus* is of seminal importance. The present findings will contribute to our understanding of the molecular mechanisms used by *S. aureus* to survive in infected cells, a key step in *Staphylococcal* pathogenicity.

## Materials and Methods

### Materials

α-MEM and D-MEN cell culture media and fetal calf serum were obtained from Invitrogen, Argentina (Buenos Aires, Argentina). H89 was purchased from LC Laboratory (Massachusetts, USA). A polyclonal rabbit anti-LC3 antibody was purchased from Sigma (Buenos Aires, Argentina). The anti-myc antibody, the anti-Rap2b antibody, and Rap2b siRNA were purchased from Santa Cruz Biotechnology (Buenos Aires, Argentina). All the other reagents were from Sigma (Buenos Aires, Argentina). The anti-phosphoCREB was kindly provided by Dr. Verónica García (Facultad de Ciencias Exactas, UBA, Buenos Aires, Argentina).

### Plasmids

pEGFP-LC3 was kindly provided by Dr. Noboru Mizushima (The Tokyo Metropolitan Institute of Medical Science, Japan). The insert encoding the LC3 protein was subcloned into the red fluorescent protein vector (pRFP, kindly provided by Dr. Philip Stahl, Washington University). Briefly, the insert from pEGFP-LC3 was cut with the Bgl II and EcoRI restriction enzymes and subcloned in the corresponding restriction sites of pRFP vector. The pGFP-Rap2B wt and pGFP-Rap2B ΔAAX were kindly provided by Dr. Mauro Torti (University of Pavia, Pavia, Italy). The plasmids pCMV myc-Epac wt and pCMV myc-ΔEpac were kindly provided by Dr. Omar A. Coso (IFIBYNE, Facultad de Ciencias Exactas, UBA, Buenos Aires, Argentina).

### Cell Culture and Transfection

CHO cells, an ovary hamster cell line, were grown in α-MEM supplemented with 10% FCS, streptomycin (50 µg/ml), and penicillin (50 U/ml). HeLa human epithelial cells were grown in D-MEM supplemented with 10% FCS, streptomycin (50 µg/ml), and penicillin (50 U/ml). For some experiments cells were incubated in starvation medium EBSS (Earle's balanced salt solution). Stably transfected CHO cells overexpressing pEGFP-LC3 were used. For some experiments CHO cells were transiently cotransfected with pRFP-LC3 and pGFP-Rap2B wt or pGFP-Rap2B ΔAAX; or with pGFP-LC3 and pCMV myc-Epac wt or myc-ΔEpac. Cells were cotransfected using Lipofectamine 2000 (Invitrogen), according to the manufacturer's instructions. Stably transfected CHO cells overexpressing pEGFP were used as control.

### Treatment with the Toxin

Transfected CHO cells were incubated for 4 hours (h) with 10 µg/ml of α-hemolysin from *S. aureus* (Sigma Aldrich; Buenos Aires, Argentina). Cells were fixed and analyzed by confocal fluorescence microscopy.

### Bacterial Strains and Growth Conditions

For infection experiments, *S. aureus* strains, wt (01016), the mutant deficient for α-hemolysin (Hla−) (01017), or the Hla(−) mutant complemented with an α-hemolysin plasmid (01018) were grown overnight at 37°C in 5 ml of LB broth with appropriate antibiotics. Bacteria were resuspended in infection medium containing 10% FCS and 20 mM HEPES, at an OD_650_ of 0.4 (∼4×10^8^ CFU). Bacteria were diluted to achieve a multiplicity of infection (moi) of 10∶1 (bacteria∶cell) in the infection medium.

### Fluorescence Microscopy

Transfected CHO cells were analyzed by fluorescence microscopy using an Olympus Confocal FV1000 (Japan) and processed with the program FV10-ASW 1.7. In some experiments, to visualize the pathogen, bacterial DNA was labeled with 45 nM TOPRO in Mowiol.

### PKA and EPAC Activation

CHO GFP-LC3 cells were preincubated 30 min with 1 mM N6,2′-O-DIBUTYRYLADENOSINE 3′:5′-CYCLIC (dbcAMP; Sigma; Buenos Aires, Argentina) or 10 µM 8-pCPT-2′-*O*-Me-cAMP-AM (8-pCPT; BioLog; Bremen, Germany), and then they were treated with 10 µg/ml of α-hemolysin for 4 h or incubated in starvation medium for 2 h in the presence of the drugs. Cells were fixed and analyzed by confocal microscopy.

### SDS-PAGE and Western Blot

CHO and HeLa cells were incubated under different conditions and lysed with sample buffer. Protein samples of a total cell lysate were run on a 10% polyacrylamide gel and transferred to Hybond-ECL (Amersham) nitrocellulose membranes. The membranes were blocked for 1 h in Blotto (5% non-fat milk, 0.1% Tween 20, and PBS), washed twice with PBS and incubated with a primary antibody anti-LC3 and a peroxidase-conjugated secondary antibody (Jackson Immuno Research, 211-032-171). Anti-tubulin (Jackson Immuno Research) was used as a loading control. The corresponding bands were detected using an enhanced chemiluminescence detection kit from Healthcare (Amersham, RPN2109) and the band was detected using Fujifilm LAS-4000.

### PKA Inhibition

CHO GFP-LC3 cells were preincubated 30 min with 10 µM H89 in the presence or absence of 1 mM db cAMP, and then they were treated with 10 µg/ml of α-hemolysin for 4 h or incubated in starvation medium for 2 h in the presence of the inhibitor. Cells were fixed and analyzed by confocal microscopy.

### Calpains Inhibition

CHO GFP-LC3 cells were preincubated 30 min with 10 µM calpeptin, in the presence or the absence of 1 mM dbcAMP. Subsequently, they were treated with 10 µg/ml of α-hemolysin for 4 h or incubated in starvation medium for 2 h. Cells were fixed and analyzed by confocal microscopy.

### CFU Determination

CHO cells were infected for 3 h with the wt strain of *S. aureus*, the mutant deficient for α-hemolysin (Hla−), or the Hla(−) mutant expressing an α-hemolysin plasmid. Infected cells were washed with PBS and lysed in water at 4°C. Lysates were diluted with PBS, plated on LB agar and incubated for 12 h at 37°C. Colonies were counted on the plate with dilutions yielding 50–100 visible colonies as previously determined [21].

### Rap2b-GTP Precipitation Assays

HeLa cells were incubated for 4 h in the presence or absence of 10 µg/ml Hla, or they were infected for 4 h with *S. aureus* wt strain or Hla (−) strain. Afterwards, cells were lysed in a GST pull-down buffer (200 mM NaCl, 2.5 mM MgCl2, 1% [v/v] Triton X-100, 10% glycerol, 1 mM phenylmethylsulfonyl fluoride, a protease inhibitor mixture [Pepstatin, Leupeptin and Trypsin inhibitor], and 50 mM Tris-HCl, pH 7.4) by sonication on ice (two times for 30 s) and used immediately. Glutathione-sepharose beads were washed twice with the GST pull-down buffer and incubated with bacterial lysates containing GST-Ral-GDS-RBD for 1 h at 4°C under constant rocking. Beads were washed twice with PBS and once with GST pull-down buffer and used immediately. Twenty µl of glutathione-sepharose containing 10 µg of the appropriate fusion protein was added to cell lysates in a total volume of 0.6 ml and incubated by rotation at 4°C for 1 h. The resin was recovered by centrifugation at 4°C (5 min at 10,000 rpm) and washed three times with ice-cold GST pull-down buffer [25]. The resin-bound fractions were resolved by SDS-PAGE, and cellular GTP-Rap2b levels were analyzed by immunoblotting as described earlier, using a primary antibody anti-Rap2b and a peroxidase-conjugated secondary antibody (Jackson Immuno Research).

### Intracellular cAMP Measurement

CHO cells were incubated 4 h with 10 µg/ml of α-hemolysin or infected 4 h with the wt strain of *S. aureus* (01016) or the mutant deficient for Hla (01017). After incubations, cells were placed on ice, washed with PBS, and 0.7 ml cold 100% ethanol was added to each well. Cells were scraped and transferred to tubes (Eppendorf), sonicated twice for 2 min, and heated for 5 min at 95 C° to destroy endogenous proteins. Afterwards, the samples were centrifuged 5 min at 10,000 rpm. Supernatants were dried and kept at −20 C°. Samples were diluted in 200 µl of 50 mM sodium acetate buffer (pH 6.0). Unknown samples and standards were acetylated and assayed by RIA using the method described by Del Punta et al. [43]. The interassay and intraassay variations of coefficients were lower than 10%.

## Supporting Information

Figure S1 **Model showing the two cAMP-dependent pathways involved in the control of autophagy and the effect of different molecules on the autophagic response induced by Hla and by** ***S. aureus*** **.** (A) Different stimuli, like bacterial infections, may modulate the levels of intracellular cAMP, either increasing or decreasing the intracellular levels of this second messenger. When the levels of cAMP are augmented, autophagy can be inhibited via two different pathways: (Left) cAMP activates PKA, which is able to phosphorylate LC3-I, avoiding its conversion to LC3-II and preventing autophagy activation. (Right) On the other hand, cAMP can activate EPAC, which through Rap2b and PLCε activation induces an IP3 increase. In turn, this IP3 increase leads to calcium release from the endoplasmic reticulum, which activates the cysteine-protease calpains. These calpains are able to cleave Atg5, inhibiting the autophagic pathway. (B) Left: Inactivation of PKA with H89 does not prevent the inhibitory effect of cAMP on the Hla or *S. aureus*–induced autophagy, suggesting that this effect occurs via a PKA-independent pathway. Right: EPAC or Rap2b overexpression is sufficient to inhibit the autophagy induced by Hla or *S. aureus*, indicating that these proteins participate in the regulation of the autophagic response induced by this pathogen. Furthermore, inhibition of calpains with calpeptin allows autophagy activation induced by Hla even in the presence of cAMP, suggesting that inactivation of calpains is necessary for this autophagic response.
(TIF)

Figure S2 **Endogenous LC3 recruitment and Atg12-Atg5 complex formation are inhibited by cAMP treatment.** (A) CHO cells were preincubated with 1 mM dybutiryl cAMP (+dbcAMP) for 30 min, and then they were treated for 4 h with 10 µg/ml of α-hemolysin (Hla), with 50 ng/µl of rapamycin (Rapa) in full nutrient media or subjected to starvation conditions (Stv) in the continuous presence of dbcAMP. Cells without any treatment were used as control (−dbcAMP). Endogenous LC3 was detected using a rabbit anti-LC3 and cells were visualized by confocal microscopy. These data are representative of three independent experiments. (B) CHO cells were incubated with complete medium in the absence (−dbcAMP) or presence of dbcAMP (+dbcAMP) and treated for 4 h with 10 µg/ml of α-hemolysin (Hla) or subjected to starvation conditions (Stv). Afterwards, cells were lysed with sample buffer and the samples were subjected to Western blot analysis using a rabbit anti-Atg12 and the corresponding HRP-labeled secondary antibody, and subsequently developed with an enhanced chemiluminescence detection kit. These data are representative of two independent experiments. The band intensities of two independent experiments were quantificated with the Adobe Photoshop program (lower panel). * *p*<0.05 (paired Student's t-test).
(TIF)

Figure S3 **Autophagic response upon Hla treatment in cells overexpressing the vectors RFP or GFP.** (A) CHO cells were cotransfected with GFP-LC3 and RFP-Vector. Twenty-four hours after transfection, they were incubated for 2 h in starvation medium (Stv) or treated for 4 h with 10 µg/ml of α-hemolysin (Hla). Cells without any treatment were used as control (Ctr). Cells were analyzed by confocal microscopy. Images are representative of two independent experiments. (B) Quantification of the percentage of cells presenting LC3 puncta upon incubation under the different conditions (*n* = 50 cells/condition). These data are representative of two independent experiments. (C) CHO cells were cotransfected with RFP-LC3 and GFP-Vector. Twenty-four hours after transfection, they were incubated for 2 h in starvation medium (Stv), with 50 ng/µl of rapamycin (Rapa) in full nutrient media, or treated for 4 h with 10 µg/ml of α-hemolysin (Hla). Cells without any treatment were used as control (Ctr). Cells were analyzed by confocal microscopy. Images are representative of three independent experiments. (D) CHO cells were transfected with GFP-Vector and incubated for 4 h with complete medium without (Ctr) or with 10 µg/ml of α-hemolysin (Hla), with 50 ng/µl of rapamycin (Rapa) or subjected to starvation conditions (Stv) for 2 h. Afterwards, cells were lysed with sample buffer and the samples were subjected to Western blot analysis using a rabbit anti-LC3 and the corresponding HRP-labeled secondary antibody, and subsequently developed with an enhanced chemiluminescence detection kit. The band intensities were quantificated with the Adobe Photoshop program (lower panel). These data are representative of two independent experiments.
(TIF)

Figure S4 ***S. aureus*** **infection after overexpression of the RFP-Vector.** (A) CHO cells were cotransfected with GFP-LC3 and RFP-Vector. Twenty-four hours after transfection, they were infected for 4 h with the wt strain of *S. aureus* (wt), the mutant deficient for α-hemolysin (Hla−), or the Hla(−) mutant expressing an α-hemolysin plasmid (Hla(−)+pHla). The nucleus and the bacteria were labeled with TOPRO as indicated in Materials and Methods and immediately visualized by confocal microscopy. Images are representative of two independent experiments. (B) Quantification of the percentage of cells presenting LC3 puncta upon incubation under the different conditions (*n* = 50 cells/condition). These data are representative of two independent experiments.
(TIF)

Figure S5 **Endogenous EPAC and Rap2b are recruited to the** ***S. aureus*** **–containing phagosomes.** CHO cells were infected for 4 h with the wt strain of *S. aureus* (wt), the mutant deficient for α-hemolysin (Hla−), or the Hla(−) mutant expressing an α-hemolysin plasmid (Hla(−)+pHla). Endogenous EPAC (upper panels) and Rap2b (lower panels) were detected using a rabbit anti-EPAC and a rabbit anti-Rap2b, respectively. The nucleus and the bacteria were labeled with TOPRO as indicated in Materials and Methods and the samples were visualized by confocal microscopy. Quantifications of the percentage of bacteria decorated with endogenous EPAC or Rap2b upon the infection are shown in right panels. These data are representative of two independent experiments.
(TIF)

Figure S6 **Endogenous EPAC and Rap2b do not colocalize with LC3.** CHO GFP-LC3 cells were infected for 4 h with the wt strain of *S. aureus* (wt), the mutant deficient for α-hemolysin (Hla−), or the Hla(−) mutant expressing an α-hemolysin plasmid (Hla(−)+pHla). Endogenous EPAC (upper panels) or Rap2b (lower panels) were detected using a rabbit anti-EPAC and a rabbit anti-Rap2b, respectively. The nucleus and the bacteria were labeled with TOPRO as indicated in Materials and Methods and the samples were visualized by confocal microscopy. These data are representative of two independent experiments.
(TIF)
