# Supplementary figures and images for: cAMP and EPAC Are Key Players in the Regulation of the Signal Transduction Pathway Involved in the α-Hemolysin Autophagic Response
Source: PLoS Pathog. 2012 May 24;8(5):e1002664. doi: 10.1371/journal.ppat.1002664 (PMC3359991; doi:10.1371/journal.ppat.1002664)

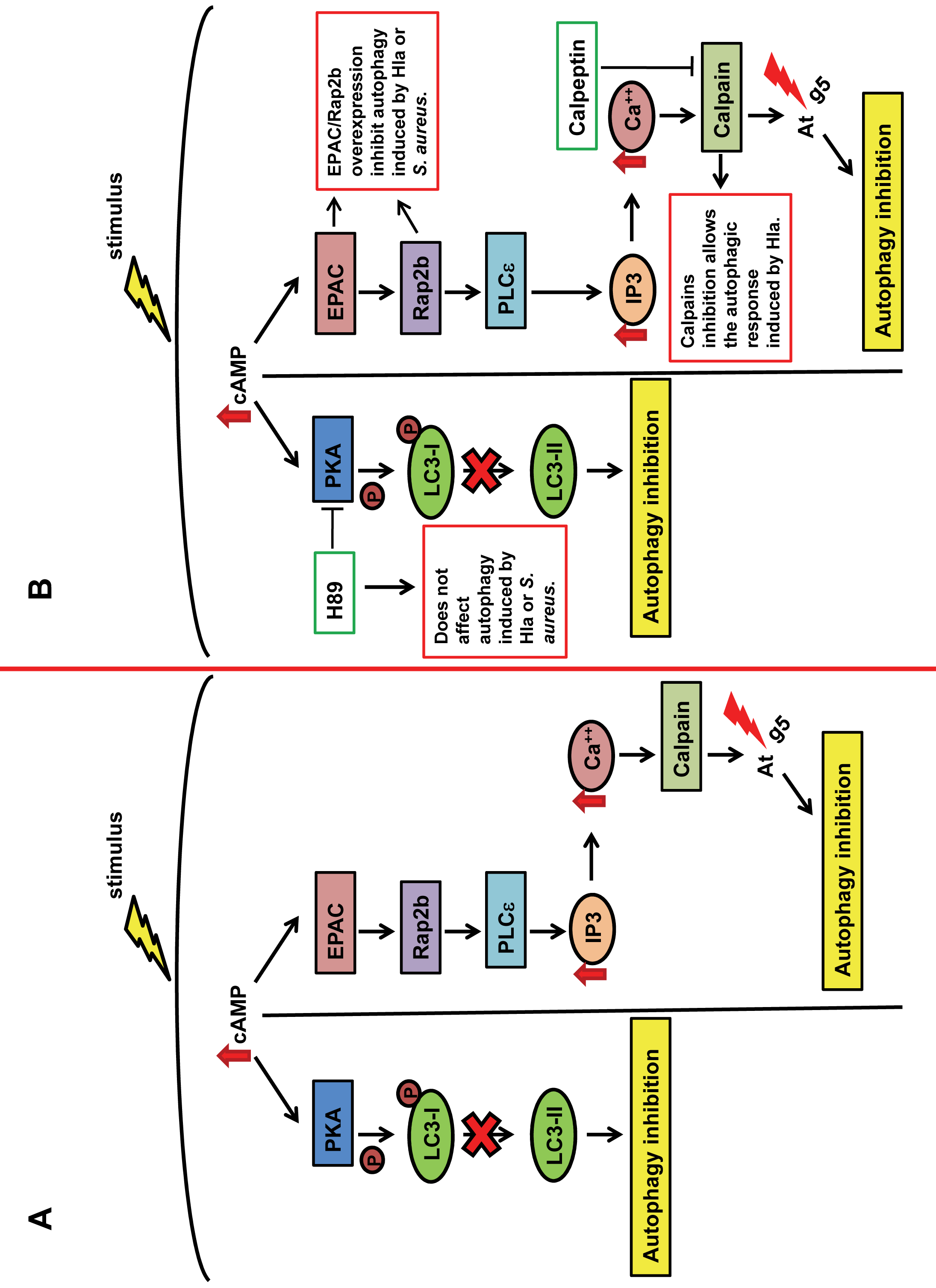

Supplement: Figure S1 — Model showing the two cAMP-dependent pathways involved in the control of autophagy and the effect of different molecules on the autophagic response induced by Hla and by S. aureus . (A) Different stimuli, like bacterial infections, may modulate the levels of intracellular cAMP, either increasing or decreasing the intracellular levels of this second messenger. When the levels of cAMP are augmented, autophagy can be inhibited via two different pathways: (Left) cAMP activates PKA, which is able to phosphorylate LC3-I, avoiding its conversion to LC3-II and preventing autophagy activation. (Right) On the other hand, cAMP can activate EPAC, which through Rap2b and PLCε activation induces an IP3 increase. In turn, this IP3 increase leads to calcium release from the endoplasmic reticulum, which activates the cysteine-protease calpains. These calpains are able to cleave Atg5, inhibiting the autophagic pathway. (B) Left: Inactivation of PKA with H89 does not prevent the inhibitory effect of cAMP on the Hla or S. aureus–induced autophagy, suggesting that this effect occurs via a PKA-independent pathway. Right: EPAC or Rap2b overexpression is sufficient to inhibit the autophagy induced by Hla or S. aureus, indicating that these proteins participate in the regulation of the autophagic response induced by this pathogen. Furthermore, inhibition of calpains with calpeptin allows autophagy activation induced by Hla even in the presence of cAMP, suggesting that inactivation of calpains is necessary for this autophagic response. (TIF) [file ppat.1002664.s001.tif]

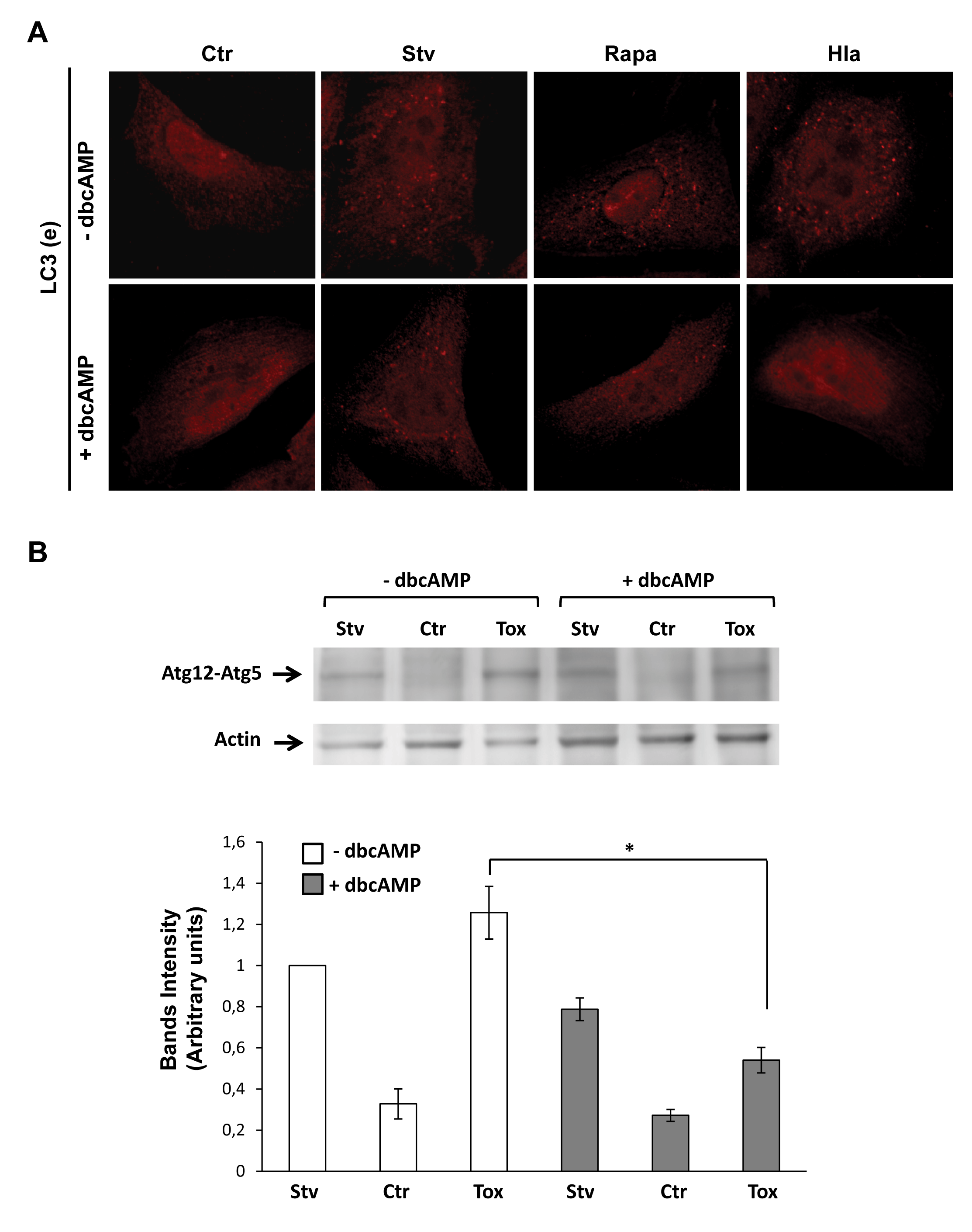

Supplement: Figure S2 — Endogenous LC3 recruitment and Atg12-Atg5 complex formation are inhibited by cAMP treatment. (A) CHO cells were preincubated with 1 mM dybutiryl cAMP (+dbcAMP) for 30 min, and then they were treated for 4 h with 10 µg/ml of α-hemolysin (Hla), with 50 ng/µl of rapamycin (Rapa) in full nutrient media or subjected to starvation conditions (Stv) in the continuous presence of dbcAMP. Cells without any treatment were used as control (−dbcAMP). Endogenous LC3 was detected using a rabbit anti-LC3 and cells were visualized by confocal microscopy. These data are representative of three independent experiments. (B) CHO cells were incubated with complete medium in the absence (−dbcAMP) or presence of dbcAMP (+dbcAMP) and treated for 4 h with 10 µg/ml of α-hemolysin (Hla) or subjected to starvation conditions (Stv). Afterwards, cells were lysed with sample buffer and the samples were subjected to Western blot analysis using a rabbit anti-Atg12 and the corresponding HRP-labeled secondary antibody, and subsequently developed with an enhanced chemiluminescence detection kit. These data are representative of two independent experiments. The band intensities of two independent experiments were quantificated with the Adobe Photoshop program (lower panel). * p<0.05 (paired Student's t-test). (TIF) [file ppat.1002664.s002.tif]

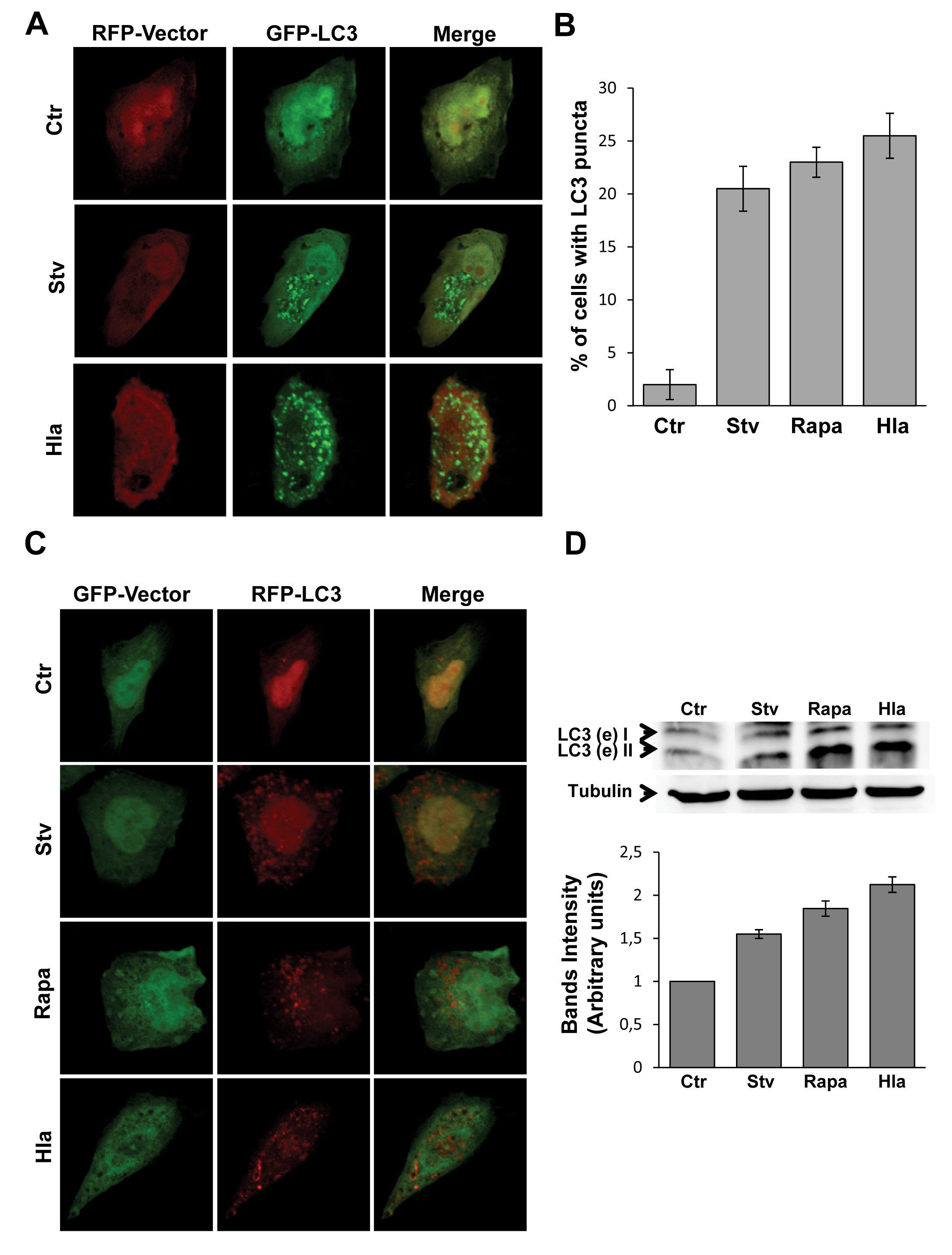

Supplement: Figure S3 — Autophagic response upon Hla treatment in cells overexpressing the vectors RFP or GFP. (A) CHO cells were cotransfected with GFP-LC3 and RFP-Vector. Twenty-four hours after transfection, they were incubated for 2 h in starvation medium (Stv) or treated for 4 h with 10 µg/ml of α-hemolysin (Hla). Cells without any treatment were used as control (Ctr). Cells were analyzed by confocal microscopy. Images are representative of two independent experiments. (B) Quantification of the percentage of cells presenting LC3 puncta upon incubation under the different conditions (n = 50 cells/condition). These data are representative of two independent experiments. (C) CHO cells were cotransfected with RFP-LC3 and GFP-Vector. Twenty-four hours after transfection, they were incubated for 2 h in starvation medium (Stv), with 50 ng/µl of rapamycin (Rapa) in full nutrient media, or treated for 4 h with 10 µg/ml of α-hemolysin (Hla). Cells without any treatment were used as control (Ctr). Cells were analyzed by confocal microscopy. Images are representative of three independent experiments. (D) CHO cells were transfected with GFP-Vector and incubated for 4 h with complete medium without (Ctr) or with 10 µg/ml of α-hemolysin (Hla), with 50 ng/µl of rapamycin (Rapa) or subjected to starvation conditions (Stv) for 2 h. Afterwards, cells were lysed with sample buffer and the samples were subjected to Western blot analysis using a rabbit anti-LC3 and the corresponding HRP-labeled secondary antibody, and subsequently developed with an enhanced chemiluminescence detection kit. The band intensities were quantificated with the Adobe Photoshop program (lower panel). These data are representative of two independent experiments. (TIF) [file ppat.1002664.s003.tif]

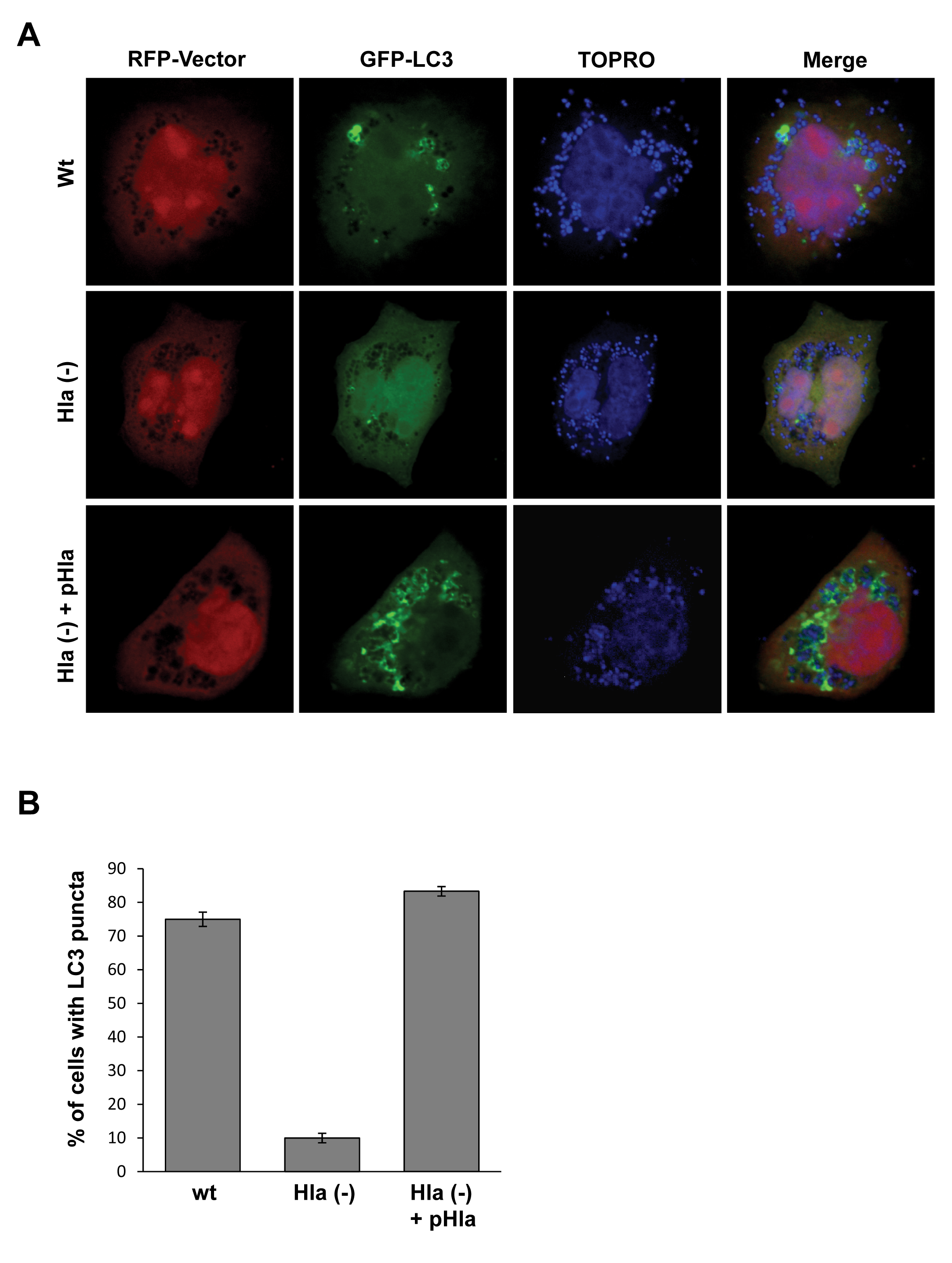

Supplement: Figure S4 — S. aureus infection after overexpression of the RFP-Vector. (A) CHO cells were cotransfected with GFP-LC3 and RFP-Vector. Twenty-four hours after transfection, they were infected for 4 h with the wt strain of S. aureus (wt), the mutant deficient for α-hemolysin (Hla−), or the Hla(−) mutant expressing an α-hemolysin plasmid (Hla(−)+pHla). The nucleus and the bacteria were labeled with TOPRO as indicated in Materials and Methods and immediately visualized by confocal microscopy. Images are representative of two independent experiments. (B) Quantification of the percentage of cells presenting LC3 puncta upon incubation under the different conditions (n = 50 cells/condition). These data are representative of two independent experiments. (TIF) [file ppat.1002664.s004.tif]

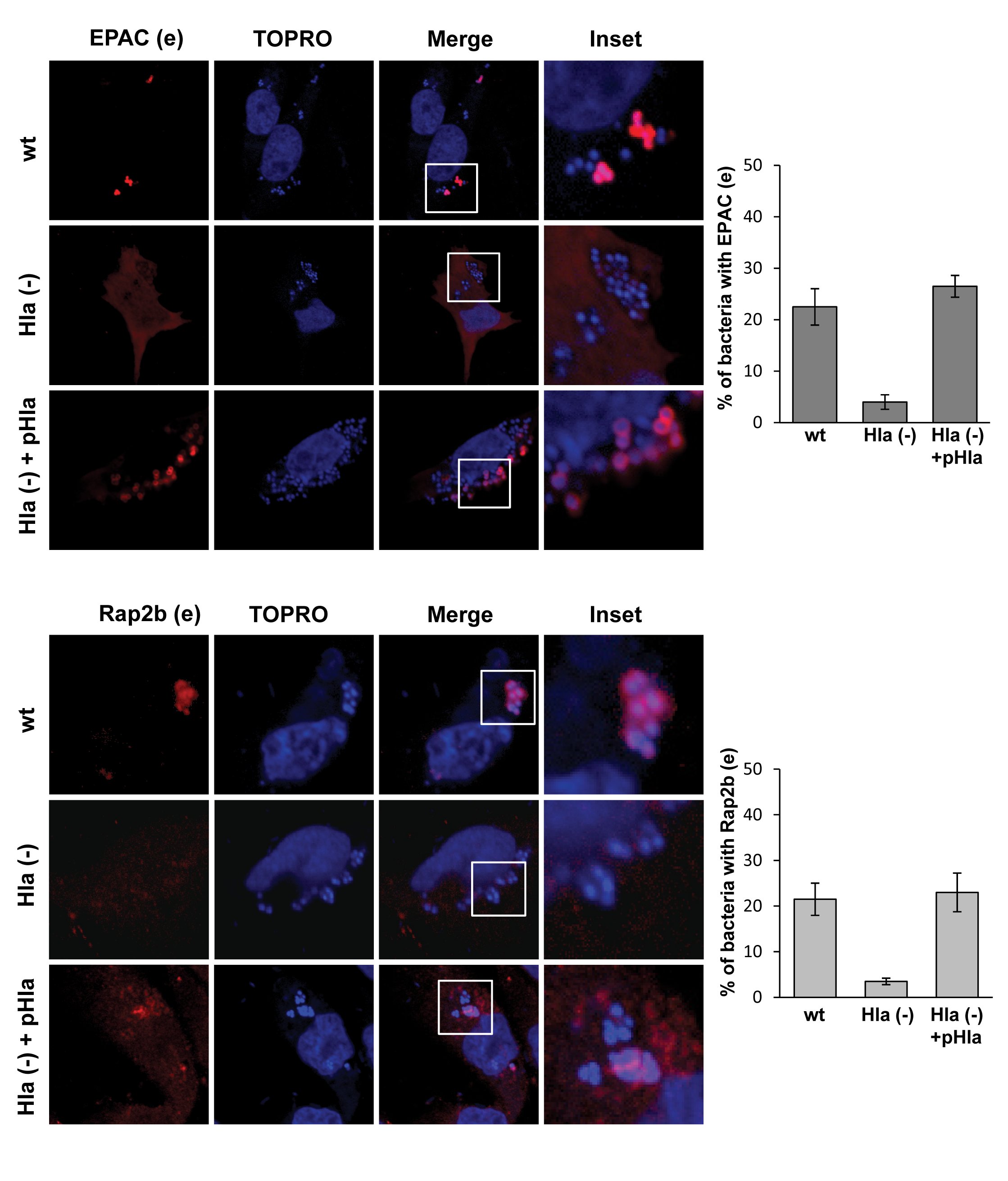

Supplement: Figure S5 — Endogenous EPAC and Rap2b are recruited to the S. aureus –containing phagosomes. CHO cells were infected for 4 h with the wt strain of S. aureus (wt), the mutant deficient for α-hemolysin (Hla−), or the Hla(−) mutant expressing an α-hemolysin plasmid (Hla(−)+pHla). Endogenous EPAC (upper panels) and Rap2b (lower panels) were detected using a rabbit anti-EPAC and a rabbit anti-Rap2b, respectively. The nucleus and the bacteria were labeled with TOPRO as indicated in Materials and Methods and the samples were visualized by confocal microscopy. Quantifications of the percentage of bacteria decorated with endogenous EPAC or Rap2b upon the infection are shown in right panels. These data are representative of two independent experiments. (TIF) [file ppat.1002664.s005.tif]

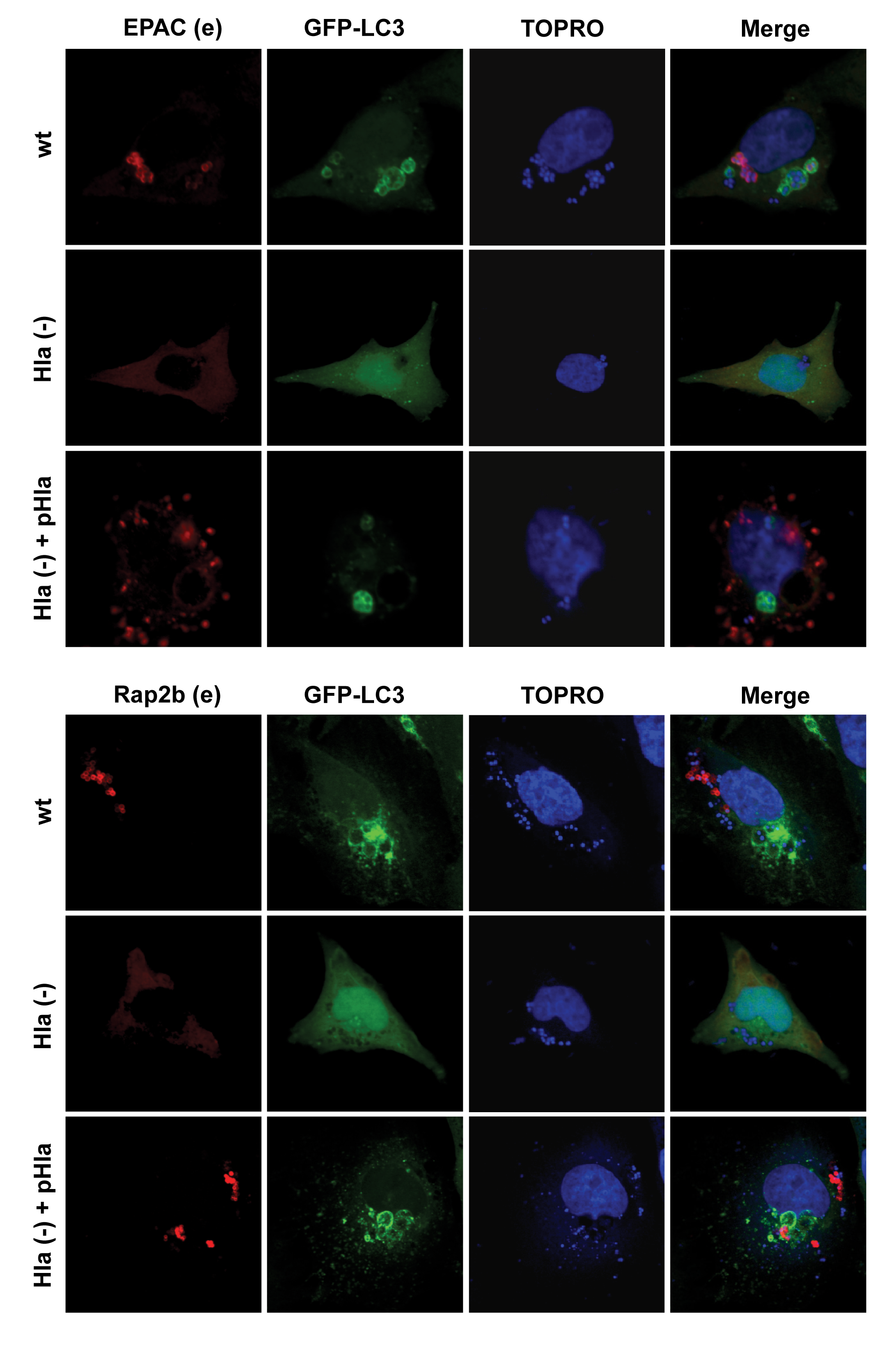

Supplement: Figure S6 — Endogenous EPAC and Rap2b do not colocalize with LC3. CHO GFP-LC3 cells were infected for 4 h with the wt strain of S. aureus (wt), the mutant deficient for α-hemolysin (Hla−), or the Hla(−) mutant expressing an α-hemolysin plasmid (Hla(−)+pHla). Endogenous EPAC (upper panels) or Rap2b (lower panels) were detected using a rabbit anti-EPAC and a rabbit anti-Rap2b, respectively. The nucleus and the bacteria were labeled with TOPRO as indicated in Materials and Methods and the samples were visualized by confocal microscopy. These data are representative of two independent experiments. (TIF) [file ppat.1002664.s006.tif]
